# Interferon epsilon is produced in the testis and protects the male reproductive tract against virus infection, inflammation and damage

**DOI:** 10.1371/journal.ppat.1012702

**Published:** 2024-12-02

**Authors:** Rukmali Wijayarathna, Eveline D. de Geus, Rosemary Genovese, Linden J. Gearing, Georgie Wray-McCann, Rajini Sreenivasan, Hiba Hasan, Monika Fijak, Peter Stanton, Daniela Fietz, Adrian Pilatz, Hans-Christian Schuppe, Michelle D. Tate, Paul J. Hertzog, Mark P. Hedger

**Affiliations:** 1 Centre for Reproductive Health, Hudson Institute of Medical Research, Melbourne, Australia; 2 Department of Molecular and Translational Sciences, School of Clinical Sciences, Monash University, Melbourne, Australia; 3 Centre for Innate Immunity and Infectious Diseases, Hudson Institute of Medical Research, Melbourne, Australia; 4 Institute of Anatomy and Cell Biology, Justus-Liebig University, Giessen, Germany; 5 Institute of Veterinary Anatomy, Histology and Embryology, Justus Liebig University, Giessen, Germany; 6 Department of Urology, Paediatric Urology and Andrology, Justus Liebig University, Giessen, Germany; Icahn School of Medicine at Mount Sinai, UNITED STATES OF AMERICA

## Abstract

The testis is a reservoir for viruses that can cause persistent infection and adversely affect male reproductive health, an observation commonly attributed to deficiencies in inducible antiviral defence mechanisms. In this study, we demonstrate that interferon-epsilon (IFNε), a type I interferon initially discovered in female reproductive epithelia, is constitutively expressed by meiotic and post-meiotic spermatogenic cells, Leydig cells and macrophages in mouse testes. A similar distribution pattern was observed in human testes. Mice lacking IFNɛ were more susceptible to Zika virus-induced inflammation and damage of the testis and epididymis compared to wild-type mice. Exogenous IFNε treatment reduced the viral infection burden in cultured human testicular cells by inducing interferon-stimulated gene expression, and reducing inflammatory gene expression and cell damage. Treatment was more effective when administered prior to infection. These data indicate a critical role for constitutively-expressed IFNɛ in limiting viral infection and inflammatory damage in the male reproductive tract.

## Author summary

Many clinically significant viruses can infect the testis, affecting male fertility, and the health of the partners and children of infected men. Crucially, many viruses are transmitted via semen, because infections can elude the immune system by hiding in the testis, presumably because of its unique “immunologically privileged” environment. However, our understanding of anti-viral defences in the testis is lacking. We found that under normal physiological conditions, Interferon epsilon (IFNε), a novel antiviral molecule initially identified in the female reproductive tract, is expressed in testicular immune cells, but more significantly in cells that develop into sperm in both mice and humans. In this paper, we show that IFNε plays a crucial role in limiting Zika virus induced damage to the male reproductive system.

## Introduction

The fact that the testis is a target for viral infections has enormous health consequences. Over one million sexually transmitted infections are acquired every day worldwide according to the World Health Organization, with more than half due to viruses, such as herpes simplex and HIV, that are currently incurable. Other emerging and endemic viruses that can infect the testis, including Zika, Mumps, Epstein–Barr virus, Cytomegalovirus, Coxsackievirus, SARS-CoV-2 and other SARS-related viruses, also pose significant risks for male reproductive health [1–5].

Viruses can infect the testis via ascending urogenital passage and via hematogenic spread during systemic virus infections. The evidence suggests that the testis can act as a viral reservoir due to its unique ‘immune privileged’ environment, which protects the immunologically foreign spermatozoa that appear in the testis long after the establishment of central immune tolerance [1,6,7]. The specialized tight junctions between epithelial Sertoli cells in the seminiferous tubules form a blood-testis-barrier that separates the adluminal compartment containing spermatogenic cells from the immune cells in the interstitial compartment. The Sertoli cells, the resident testicular macrophages which have an anti-inflammatory phenotype, and secreted immunoregulatory molecules such as interleukin-10, testosterone and activin, contribute to the maintenance of immune privilege [6,8]. The spermatogenic cells, in particular, are deficient in their ability to produce classical inducible type I interferons and interferon-stimulated genes required for antiviral responses [1,6,9–12]. Importantly, viruses that take advantage of this specialised immunological milieu can persist in the testis. The Sertoli cells, which provide nutrition and protection to developing spermatogenic cells, the spermatogenic cells themselves, and testosterone-producing Leydig cells commonly act as reservoirs for persistent viruses [7,10,13,14].

Crucially, asymptomatic men can transmit these viruses via the semen, compromising partner and offspring health [15,16]. This also poses important practical and ethical implications for assisted reproductive therapies and fertility treatments. Studying natural anti-viral defences in the male reproductive tract is essential to better understand mechanisms of viral persistence in the testis and to aid the development of specific therapies targeting testicular virus infections.

Interferon-epsilon (IFNε), an anti-viral cytokine initially identified in the female reproductive tract epithelium, plays a protective role against sexually transmitted viruses in the female [17,18]. Although a member of the type I interferon family, its constitutive expression and dependence on hormonal support by oestrogen and progesterone distinguishes it from classical inducible type I interferons [18–20]. Importantly, the role of IFNε in the male reproductive tract is largely unknown. Although IFNε mRNA was reported to be expressed in the mouse testis [18,21], the specific testicular cell types that express IFNε and the functional role of IFNε in the male reproductive tract have not been studied.

In this study, we aimed to investigate the cellular production sites of IFNε in the male reproductive tract, specifically in the testis (site of sperm and testosterone production), the epididymis (site of sperm maturation and storage) and the vas deferens (conduit between the epididymis and urethra). We also aimed to define the contribution of IFNε to testicular anti-viral defence, using the Zika virus to model persistent virus infections of the testis. Zika is a mosquito-borne flavivirus that can also be sexually transmitted up to one year after infection by asymptomatic men, leading to a range of adverse pregnancy outcomes, including abortions, still births, and congenital abnormalities [22–24].

Our data show that IFNε is constitutively expressed in murine and human testicular macrophages, Leydig cells and meiotic and post-meiotic spermatogenic cells, in the absence of infection. IFNε was also detected in human testicular interstitial fluid and seminal plasma. Significantly, we discovered that the lack of IFNε exacerbates the severity of Zika virus infection in mice leading to orchitis and epididymal fibrosis. Moreover, we showed that exogenous IFNε has prophylactic and therapeutic protective effects on Zika-infected human Sertoli cells, which appear to be the principal target cells for IFNε. IFNε conferred antiviral protection by inducing interferon-stimulated genes and by reducing proinflammatory cytokines. Overall, this study indicates that endogenous production of IFNε by the testis plays an essential role in anti-viral defence by limiting the consequences of virus infection in an organ that is relatively deficient in inducible anti-viral defences.

## Materials and methods

### Ethics statement

All animal experiments were approved by the Monash Medical Centre Animal Ethics Committee and the Monash Animal Research Platform Animal Ethics Committee and were performed in strict accordance with the National Health and Medical Research Council Code of Practice for the Care and Use of Animals for Experimental Purposes.

The study using human patient samples was approved by the Ethics Committee of the Faculty of Medicine, Justus Liebig University, Giessen (AZ 26/11) in 2011, and patients were recruited from June 2011 onwards. All patients were counselled preoperatively, and written informed consent was obtained [25].

### Animals

Male C57BL/6J wild-type (WT) mice between 5–180 days of age were purchased from Monash Animal Services. *Ifne*^-/-^ and *Ifnar1*^-/-^ mice on a C57BL/6J background were maintained in the Monash Medical Centre Animal House under standard specific pathogen-free (SPF) conditions. Animals used for Zika infection experiments were transported to the Monash Animal Research Laboratories and maintained for a minimum of one week under standard housing conditions prior to experimental procedures.

### Human patient samples

Testicular biopsies and testicular interstitial fluid were collected from infertile men aged 29–50 years suffering azoospermia and undergoing testicular surgeries for sperm retrieval at the Dept. of Urology, Pediatric Urology and Andrology, University Hospital, Justus-Liebig University, Giessen, Germany [26]. The andrological work-up comprised a structured medical history, clinical and ultrasound examination, including measurement of testicular volume, blood sampling for sex hormone profiles, genetic testing for karyotype, AZF deletions and CFTR gene mutations. Semen analysis was performed according to WHO recommendations, including peroxidase-positive leukocyte counts and seminal polymorphonuclear (PMN) elastase as routine parameters [5,27,28].

Testicular biopsies from 26 patients were examined, as reported previously [29]. The samples were either fixed in Bouin’s solution and embedded in paraffin (for histological assessment and immunofluorescence staining) or snap-frozen (for RNA isolation) [26]. Testicular damage ranged from normal spermatogenesis, quantitatively reduced but qualitatively preserved spermatogenesis (mild hypospermatogenesis), to spermatogenic cell aplasia (the Sertoli cell only [SCO] phenotype), and total tubular atrophy.

Testicular biopsies with intact spermatogenesis and no evidence of leukocyte infiltration were used for immunofluorescence localisation of IFNε in this study. qRT-PCR to detect *IFNE*, *IFNB1*, *IFNAR1* and *IFNAR2* were also performed on ‘Normal’ biopsy samples exhibiting intact spermatogenesis with no evidence of leukocyte infiltration, and compared to testicular biopsies showing various degrees of impaired spermatogenesis (moderate to severe hypospermatogenesis, spermatogenic arrest [SA], SCO phenotype, or mixed testicular atrophy [MTA]) and histological signs of inflammation, including focal leukocytic infiltrates in the interstitial compartment (‘I + WBC’ group).

IFNɛ protein was measured using an ELISA assay in human testicular interstitial fluid obtained from men separated into three categories based on testicular histology. These groups were, (1) MTA (testes contain areas with intact spermatogenesis next to various degrees of tubular damage), (2) SCO phenotype, and (3) SA (spermatogenesis is initiated, but no mature sperm are produced due to developmental arrest).

Human seminal plasma samples were obtained with informed consent from patients with normal sperm parameters (normozoospermia), positive test results for membrane-bound sperm auto-antibodies, or elevated seminal leukocyte counts (leukocytospermia) attending the Andrology Clinic (Monash Medical Centre, Clayton) or Monash IVF Clinic (Epworth Hospital), and processed as previously described [30].

### Mouse testicular somatic cell type isolation

Individual testicular cell types were isolated from testes of 42–44 day old WT mice (5 mice per cell prep) using established methods, as described below. Testes were dissected out into sterile PBS and decapsulated. The seminiferous tubules were teased apart gently, to minimize tubular damage and subsequent spermatogenic cell contamination. Isolated seminiferous tubules were separated from interstitial tissue fragments and free spermatogenic cells by sedimentation at unit gravity for 10 minutes, with two further washes with PBS to remove residual interstitial cells and free spermatogenic cells in the supernatants.

The supernatants were pooled and total interstitial cells (Leydig cells, macrophages, mesenchymal cells) were separated from the less-dense free spermatogenic cells by Percoll density gradient centrifugation [31]. Mouse Leydig cells and macrophages form aggregates due to tight intercytoplasmic junctions, which can persist even after isolation, and were not further separated in this study.

Sertoli cells were isolated using selective adherence to *Datura stramonium* agglutinin (DSA)-(Sigma) coated cell culture dishes, as previously described [32–34]. Briefly, the seminiferous tubules that were collected after the supernatant was removed were digested with a collagenase/hyaluronidase cocktail. Cells were plated at a concentration of 1×10^6^ cells/well in DSA-lectin coated 24-well culture plates containing 1 ml Dulbecco’s modified Eagle’s medium (Gibco, USA). After a 24 hour attachment period and washing to remove unattached spermatogenic cells and debris, Sertoli cells were cultured for a further 3 hours at 37°C before harvesting for mRNA analysis.

### Mouse spermatogenic cell type isolation

Spermatocytes, round spermatids and cytoplasts containing residual bodies were isolated from 8 adult (44 day old) WT mice by centrifugal elutriation, as previously described [35]. Briefly, the decapsulated testes were dissociated using collagenase and trypsin and the cell suspension was fractionated using a Beckman JE 5.0 rotor in a standard 4 ml chamber with a starting speed of 3000 rpm and flow rate of 13.5 ml/min. Cell fractions were collected at 3000 rpm, flow rate 31.3–40.7 ml/min (round spermatids), 2000 rpm, 30.2 ml/min (small pachytene spermatocytes), 2000 rpm, 51 ml/min (large pachytene spermatocytes), and 3360 rpm, 31 ml/min (cytoplasts). Cells were characterised by established morphological criteria, with 85–90% purity.

### Virus

Low passage Zika virus Puerto Rican strain PRVABC59 was propagated in in Vero E6 cells. Infectious virus stock titres were determined by plaque assay on Vero E6 cells [17].

### Mouse model of testicular infection of Zika virus

8–10 week old WT, *Ifne*^*-/-*^ and *Ifnar1*^*-/-*^mice were administered a single intraperitoneal (i.p.) injection of the Puerto Rican strain PRVABC59 of the Zika virus (5 x 10^5^ PFU) [17,36]. Controls received a similar volume (200 μl) of Phosphate Buffered Saline (PBS) i.p. WT and *Ifne*^*-/-*^ mice were culled at 3, 7 and 21 days post-infection. *Ifnar1*^*-/-*^ mice were only examined at 3 and 7 day timepoints due to ethical concerns, because they develop neurological disease 10 days post-infection [36]. Cardiac blood, reproductive tract tissues, testis draining lymph nodes (lumbar aortic and inguinal lymph nodes), and the spleen were collected for analysis.

### Human testicular cells and cell lines

Primary Sertoli cells [37,38] isolated from the adult human testis (HSerc) that were cryopreserved at passage one were purchased from ScienCell Research Laboratories, USA (Lot # 14286, Catalog #4520). Cells were grown in Sertoli Cell Medium (ScienCell Cat. #4521) at 37°C in 5% CO_2_, and passaged at 95% confluency (~ every 3 days) using mild trypsinization as per company recommendations. Immunofluorescence staining and mRNA expression of key marker genes (e.g., *SOX9*, *INHA*, *KRT18*) were used to validate these cells as human Sertoli cells [39]. Cells grown up to passage 4 were used in all experiments.

Primary Leydig cells [40] isolated from the testes of a 25-year-old healthy human male and cryopreserved at passage one was purchased from ScienCell Research Laboratories, USA (Lot#33247, Catalog #4510). Cells were grown in Leydig Cell Medium (ScienCell Cat. #4511) at 37°C in 5% CO_2_, and passaged at 90% confluency (~ every 3 days) using mild trypsinization. Immunofluorescence staining and mRNA expression of key marker genes (e.g., *CYP11A1*, *CYP17A1*, *INSL3*) were used to validate these cells as human Leydig cells. Cells grown up to passage 2 were used in all experiments.

The TCam-2 human seminoma-derived cell line, originally from Prof. S. Kitazawa, Japan [41–43], was kindly provided by Prof. Kate Loveland, Australia. The cells were grown in RPMI1640 medium (Gibco) containing penicillin/streptomycin (Gibco) and 10% foetal calf serum (Quantum Scientific) at 37°C with 5% CO_2_ in air. Cells were passaged at 90% Confluency (~ every 3 days) using mild trypsinization. Cells grown up to passage 4 were used in all experiments.

### Human cell line infection and treatment studies

To assess the response of human testicular cell types to virus infection, cells grown in 24-well plates up to 70% confluency were infected with Zika virus (strain PRVABC59) at a multiplicity of infection (MOI) of 5 or 10. One hour after incubating the cells with the virus at 37°C in 5% CO_2_, the cells were washed with media to remove the virus. Cells were cultured for 8, 12, 24 and 48 hours post-infection, with no further media changes. Culture media was collected for plaque assays and protein immunoassays, and cells were harvested and snap frozen for cellular mRNA and Zika virus RNA (vRNA) expression analysis. Uninfected cells cultured in parallel were used as controls for each timepoint.

For studies assessing the response of uninfected cells to exogenous IFNε, cells cultured in 24-well plates up to 70% confluency were treated with 100 IU recombinant human IFNɛ (rh IFNɛ) for 12, 24 and 48 hours. Cells and media were harvested at each timepoint.

To assess the therapeutic and prophylactic effects of exogenous IFNε, cells were treated with 100 IU (rh IFNɛ) 12 hours before infection (prophylactic-IFNɛ) or 1 hour after infection (therapeutic-IFNɛ), or diluent buffer alone (controls). Cells were infected with Zika virus (strain PRVABC59) at a multiplicity of infection (MOI) of 5 or 10. Cells and media were harvested 24 hours or 48 hours post-infection for RNA-seq, qPCR, and plaque assays.

All cell culture experiments were performed in triplicate wells and repeated three times. Each individual data point in the graphs represent the average of three technical replicates per culture round.

### Recombinant human IFNɛ (rh IFNɛ)

Human recombinant IFNɛ protein (rh IFNɛ) was generated in-house as previously described [44].

### Plaque assay

Media samples collected from cell cultures were immediately snap frozen on dry ice and stored at −80°C until ready for use. As described previously [17], Vero E6 cells grown in 12-well plates up to 90% confluency were infected with 250 μl of media samples serially diluted in DMEM and 10% FCS, for 1 hour at 37°C. The inoculum was removed, and cells were covered with 1 mL overlay of complete media containing 1.5% (w/v) carboxymethylcellulose (CMC) (Sigma). Assay plates were returned to culture for 5 days. Cell monolayers were then fixed with 10% formalin for 1 hour at room temperature. The plates were washed with MilliQ water and plaques were visualised using the crystal violet stain. Plaques were counted, and virus infectivity expressed as plaque-forming units (PFU) per ml.

### Histology

Bouin’s-fixed testes and epididymides from mice, and human testis biopsy samples were paraffin embedded and sectioned at a thickness of 5 μm. Tissues were stained using haematoxylin and eosin (H&E), Periodic Acid-Schiff (PAS), and Masson’s Trichrome techniques. Histological assessment of stained sections, including morphometric analysis, was performed using an Olympus BX50 microscope (Olympus, New York, USA) and Cellsens dimensions software 1.7.1 (Olympus).*Immunofluorescence*

IFNɛ protein was detected in mice and human tissues as described previously [20,45], with minor modifications. Briefly, heat-induced epitope retrieval was performed using a Biocare decloaking chamber in citrate buffer (pH 6.0) at 110°C for 5 minutes under pressure. Sections were incubated with 10% donkey serum (Vector Laboratories) in PBS/0.05% Tween-20 to block non-specific staining. Sections were incubated overnight at 4°C with the anti-IFNɛ antibody (Clone HE70, generated in-house, raised against recombinant human IFNɛ protein, but also cross reacts with the mouse) at a concentration of 10 μg/ml. Following a 1 hour incubation with Alexa Fluor 647 donkey anti-mouse IgG (Invitrogen), slides were counterstained with 4′,6-diamidine-2′-phenylindole dihydrochloride (DAPI; Molecular Probes) and coverslipped with Fluorescence mounting medium (Agilent). Slides were scanned at 20× magnification using an VS120 slide scanner (Olympus). Snapshots were taken with the OlyVIA v2.9 software (Olympus).

### Histopathological damage scoring

Histopathological scoring of testicular damage in Zika infected mouse tissues compared to controls were carried out using a scoring method modified from Nicolas et al, 2016 [46] as described in S2 Table. PAS stained sections, together with Caspase and F4/80 immunostained sections were blinded and graded by two investigators. Three consecutive histological sections per testis were used for scoring in a total of 8 mice per experimental group, and scored between 0 to 12 (0 = no damage, 12 = highest degree of damage) according to the extent of interstitial oedema and vascular congestion, number of F4/80+ cells as well as the presence of F4/80+ cell aggregate numbers, and caspase+ cell numbers in the seminiferous tubules. The epididymal histopathology scoring was performed using a scoring system modified from Wijayarathna et al, 2020 [47], as detailed in S3 Table. Masson’s Trichrome stained slides were blinded and scored by two investigators. Three longitudinal histological sections of the entire epididymis per mouse were used for scoring a total of 8 mice per experimental group. This score was combined with qRT-PCR values for cauda epididymal *Tnf* and *Il6* for each animal to generate a final histopathological score for the epididymis, with 0 = no damage, 4 = highest degree of damage.

### In-situ hybridization

Single Plex in-situ hybridization was carried out on 5 μm formalin-fixed, paraffin-embedded mouse testis sections using the RNAScope 2.5 (brown) assay (Advanced Cell Diagnostics) as described previously [48]. Four mice per experimental group were examined, and six sections per testis were stained, representing the mid region and a polar region of the testis. Deparaffinized tissues were treated with 3% Hydrogen Peroxide and incubated with target retrieval reagents for 15 minutes, followed by 30 minute Protease Plus pre-treatment. Next, the Zika virus probe (Advanced Cell Diagnostics Catalog No: 467871) was added to the sections and incubated in a hybridization oven for 2 hours at 40°C. Sections bearing positive and negative control probes (Advanced Cell Diagnostics) were also included. Following this, sections were incubated with AMP 1–6 for the recommended durations. The signal was detected using diaminobenzidine (DAB), slides were counterstained using Haematoxylin. Samples were mounted on glass slides with DPX. Slides were scanned at 40x magnification using an Aperio Scanscope (Leica Biosystems). The staining intensity was quantified with the Aperio ImageScope software (Leica Biosystems).

### Immunoassays

IFNε was measured in human testicular interstitial fluid samples and seminal plasma samples using an in-house human IFNε sandwich ELISA. 250 μl of each sample was run in duplicate. 25 μl of testicular interstitial fluid per patient were pooled from 4 patients in each category to obtain a sufficient sample size for the assay. The assay sensitivity was 3.9 pg/ml. Activin A was measured in cell culture media using a specific activin A ELISA employing antibodies supplied by Oxford Brookes University, as described previously [49]. The assay sensitivity was 8.671 pg/ml (Intra Assay CV%: 7.7%, Inter Assay CV%: 8.1%).

### Flow cytometry

Mouse lumbar and inguinal lymph node cells were collected by squeezing through a 70 μm filter. Viability staining was performed with LD blue (Thermo Fisher), and Fc receptors were blocked with anti-mouse CD16/32 (eBioscience). Surface staining was performed using anti-CD45-BV510 (Clone 30-F11; BD), anti-CD3-FITC (Clone 145-2C11; Tonbo Bioscience), anti-CD4-BV785 (Clone RM4-5; eBioscience), anti-CD8-APC (Clone 53–6.7; BD), anti-CD11b-SB702 (Clone M1/70; eBiosciences), anti-CD69-PE (Clone H1.2f3; BD), anti-PD-1-PECy7 (Clone J43; eBioscience), anti-MHC II-BUV737 (Clone M5/114.15.2; ThermoFisher), and anti-CD11c-PE Cy7 (N418; eBioscience), anti-F4/80-SB600 (Clone BM8; ThermoFisher), anti-CD80-PE (Clone 16-10A1; Biolegend), anti-CD86-BV421 (Clone GL-1; Biolegend), Fluorescence-minus-one samples were used to assist in analysis. Samples were analyzed using a 5 laser Cytek Aurora and FlowJo V10 software (BD). Graphs were prepared with GraphPad Prism 9 (Graph Pad Software).

### qRT-PCR

**Fig 1:**
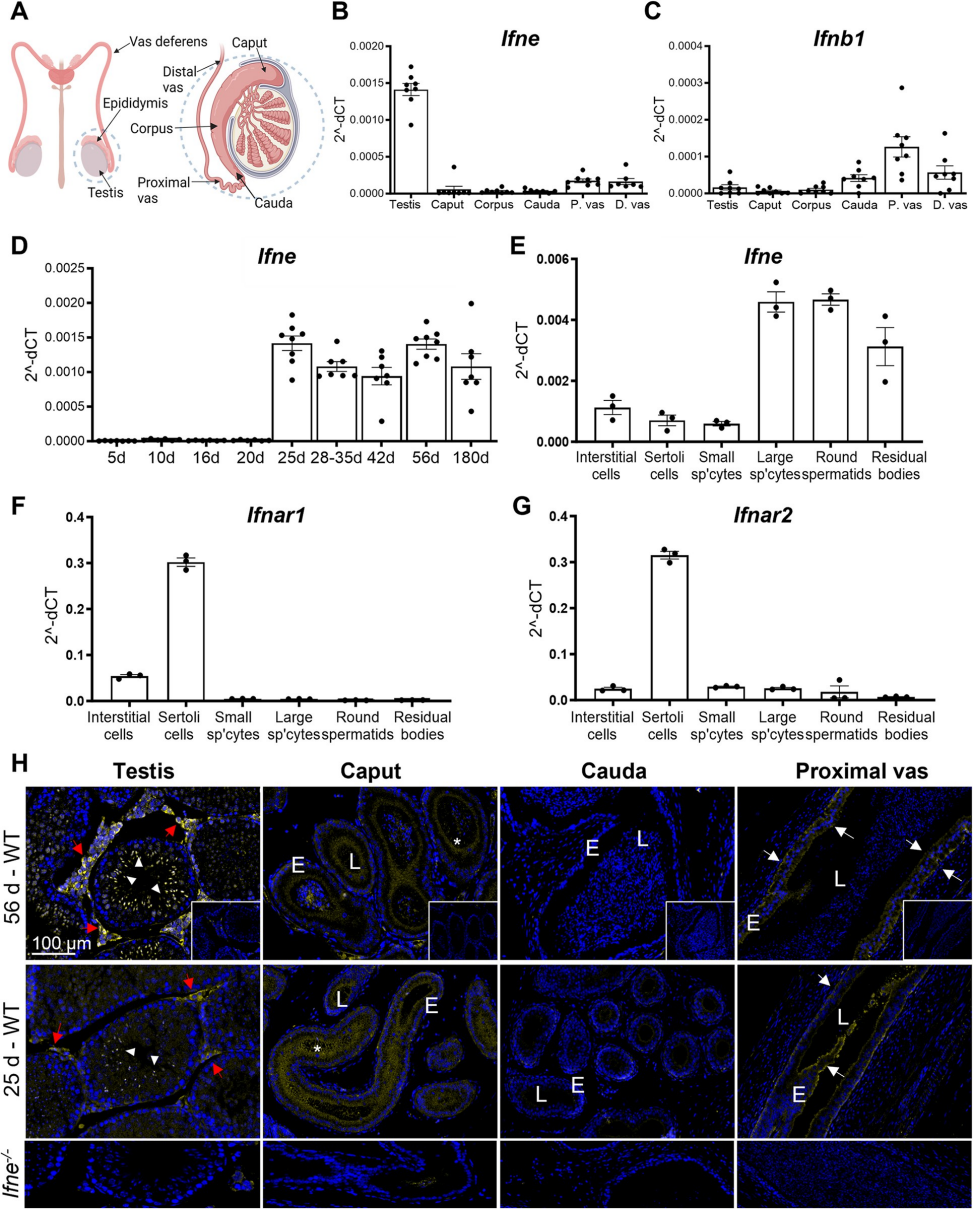
IFNɛ is constitutively expressed in the mouse male reproductive tract. (A) Schematic diagram of the anatomy of the male reproductive tract. (B) *Ifne* and (C) *Ifnb1* mRNA expression in the testis, epididymis (caput, corpus and cauda regions) and vas deferens (P. vas = proximal and D. vas = distal region of vas deferens) in 56-day-old mice (n = 8), (D) *Ifne* mRNA expression in the testis in mice aged 5–180 days (n = 6–8 per group). (E) *Ifne* and (F) type I interferon receptor *Ifnar1* and (G) *Ifnar2* mRNA expression in testicular cell types isolated from 44-day-old WT mice (n = 3 cell preps per cell type, Sp’cytes = spermatocytes). All mRNA expression data is relative to the reference gene *Rplp0* (2^-dCt = relative copy number). (H) IFNε localisation in 56- and 25-day-old WT adult mouse male reproductive tract, compared to that of 56-day-old *Ifne*^-/-^ mice using an anti-human IFNɛ antibody validated for mouse. Representative images from n = 4 mice. Inset: Isotype control. Yellow: IFNɛ. Blue: DAPI. Scale bar: 50 μm. Red arrows: Leydig cell and macrophage clusters. Arrowhead: post-meiotic spermatogenic cells. White arrows: Epithelial staining in vas deferens. Asterisk: stereocilia layer of the epididymal epithelium. E = epithelium of epididymis and vas deferens. L = lumen. The Biorender software was used to create Fig 1A.

Total cellular RNA was extracted from human and mouse tissues and cell lines using the RNeasy mini kit (QIAGEN GmBH, Hilden, Germany). Following on-column DNAse treatment, cDNA was synthesized using the Superscript III first-strand synthesis kit (Life Technologies, Carlsbad, CA, USA) from 400 ng total RNA per reaction. mRNA expression was analysed using SYBR green in a final reaction volume of 10 μL. qRT-PCR was carried out using the QuantStudio 6 Real-time PCR System (Applied Biosystems) at the Hudson Genomics Facility in Clayton, Australia. The average threshold value (Ct) of each target gene was normalized to that of the reference gene ribosomal protein, large, P0 (*Rplp0*). To compare gene expression across different organs or different cell types, and to compare Zika viral RNA in untreated vs treated cell cultures, the ΔCt method was used. These data were presented as 2^-dCt (relative copy number) values (Figs 1, 8A, S7A, S8A, and S8B). For all other gene expression data the ΔΔCt method [50] was used to quantify fold change relative to a control sample, and presented as relative mRNA expression. Primers used are listed in S1 Table.

### RNA sequencing

RNA-seq transcriptomics of Zika-infected primary human Sertoli cells treated with either prophylactic or therapeutic IFNε was performed by the Hudson Genomics Facility (Melbourne, Australia). Total RNA concentration was quantified by Qubit (Invitrogen) and quality checked by Bioanalyzer (Agilent) or capillary electrophoresis (BiOptic QSep100). Libraries were generated using an in-house multiplex RNA-seq method (version 01/09/2021) and were prepared using 25 ng of total RNA input. An 8 bp i7 sample index and a 10 bp unique molecular identifier (UMI) were added during initial poly(A) priming and pooled samples were amplified using a template-switching oligonucleotide. Illumina P5 was added by tagmentation by Nextera transposase and PCR and Illumina P7 was added by PCR. The library was designed so that the forward read (R1) used the standard R1 primer to sequence the cDNA in the sense direction for transcript identification. Samples were prepared as two libraries; the index read (I1) used the standard i7 primer to sequence directly into the sample index and then the 10 bp UMI, and an additional index read (I2) sequenced the i5 library index. Sequencing was performed on a NextSeq 2000, as per the manufacturer’s instructions (Illumina). Samples were run on a P3 50 cycle kit (R1 up to 61 bp; I1 18 bp; I2 8 bp). On-board denaturing and clustering were done using 900 pM of library pool (Illumina Protocol 1000000109376 v3 Nov2020). Base calling was performed using Dragen BCLConvert (v3.10.11).

### RNA sequencing analysis

RNA-seq read alignment and UMI counting was performed in R (v4.1.2) [51]. The scPipe package (v1.14.0) [52] was employed to process the data. Multiplex RNA-seq FASTQ files had already been demultiplexed and contained the I1 index and UMI sequences in the read header, but headers were reformatted using awk for compatibility with scPipe.

Read alignment was performed using the RSubread package (v2.6.4) [53]. Indices were built using the Ensembl *Homo sapiens* GRCh38 primary assembly genome files and alignments were performed with default settings. Aligned reads were mapped to exons using the sc_exon_mapping function with the Ensembl Homo sapiens GRCh38 v110 GFF3 genome annotation files. BAM files were processed and reads mapping to exons were associated with each individual sample using the sc_demultiplex function, taking the UMI into account, and an overall count for each gene for each was sample was generated using the sc_gene_counting function (with UMI_cor = 1).

Additional gene annotation was obtained using the biomaRt package (v2.50.1) [54,55] and a DGEList object was created with the counts and gene annotation using the edgeR package (v3.36.0) [56]. A design matrix was constructed grouping samples by treatment regime (IFNε or buffer; therapeutic or prophylactic), viral dose (MOI 5 or 10) and infection time (24 or 48 hours).

Lowly expressed genes were removed using the filterByExpr function and normalisation factors were calculated using the TMM method [57]. Counts were transformed using the voom method [58] and a linear model was fit using the edgeR voomLmFit function.

Differential gene expression analyses were performed using the limma (v3.50.0) [59] package. Groups were compared using the contrasts.fit function and moderated *t*-statistics were calculated using the eBayes function [60]. Differentially expressed genes were determined using a false discovery rate (FDR)-adjusted p-value < 0.05.

For plotting gene expression values, log_2_ counts per million (CPM) expression values were calculated using the edgeR cpm function. Relative log_2_ CPM expression values were calculated for each gene by subtracting the average log_2_ CPM value of the 24 hour prophylactic buffer MOI 5 group. For log_2_ fold changes, top genes were selected by recalculating the moderated *t*-statistics using the more stringent treat function with a twofold cut-off [61]. Genes selected had an adjusted p-value < 0.05 in at least one IFNε treatment versus its matched buffer control (significance indicated by an asterisk). Heat maps were made using the pheatmap package (v1.0.12), with scales truncated at ±5.

Hallmark [62] and Reactome [63] gene set collections were obtained from the Broad Institute Molecular Signature Database [64], via the msigdbr package (v7.4.1). Gene set testing was performed using the cameraPR function [65] from the limma package on the moderated *t*-statistics. Test results were presented as heat maps showing the average log_2_ fold changes of genes in each set (rows) for relevant comparisons (columns), with scales truncated at ±2. The significance of each gene set was indicated by text as the −log_10_ FDR-adjusted p-value threshold (p < 0.05 denoted as *, p < 0.01 denoted as 2, p < 0.001 denoted as 3 and so on).

### Statistical analysis

GraphPad Prism 9 (GraphPad Software, La Jolla, CA, USA) was used for statistical analysis. Significance for parametric data were determined using either unpaired two-tailed *t*-test, one-way analysis of variance (ANOVA) or two-way ANOVA (with appropriate post hoc tests as indicated in figure legends). Non-parametric data were assessed using Mann–Whitney U test. Differences were considered significant if the P value was < 0.05, and significance is indicated as *P < 0.05, **P < 0.01, ***P < 0.001, ****P < 0.0001. All data are expressed as mean ± standard error of mean (SEM), unless otherwise indicated.

## Results

### IFNɛ is constitutively expressed in the murine male reproductive tract

IFNɛ was constitutively expressed in the male reproductive tract of adult C57BL/6 WT mice in the absence of infection (Fig 1B). *Ifne* mRNA expression was highest in the testis (Ct values of 23–24), but negligible in the caput, corpus, and cauda regions of the epididymis (Ct values of 35 and above). The vas deferens showed significant, but relatively low expression of *Ifne* with Ct values of 32–33 in both the proximal and distal segments. By contrast, *Ifnb1*, an inducible type I interferon, showed low expression in the testis and proximal epididymis (Ct values of 35 and above), and highest expression in the cauda and vas deferens (Ct values of 32–33) (Fig 1C).

The developmental timeline of IFNɛ mRNA expression was measured in the testes of mice aged 5–180 days (Fig 1D). *Ifne* expression first appeared between day 20–25 in the mouse testis, coinciding with sexual maturation and the onset of meiosis of spermatogenic cells. To identify which testicular cell types expressed IFNɛ, mRNA expression was quantified in individual testicular cell types isolated from 44-day-old WT mice (Fig 1E). *Ifne* expression was highest in the most mature spermatogenic cell fractions: large spermatocytes, round spermatids, and residual cytoplasmic bodies (Ct values ~26–27). Interstitial cells, which included both testicular macrophages and Leydig cells, showed moderate expression (Ct: 28). Sertoli cells and small (less advanced) spermatocytes showed low *Ifne* expression (Ct values 30 and 34 respectively). However, expression of the genes encoding the type I interferon receptors, *Ifnar1* and *Ifnar2*, was considerably higher in Sertoli cells (Ct: 20) than in interstitial cells (Ct 22 and 24), while spermatogenic cells and the residual cytoplasmic bodies had relatively low expression (Ct values ~27–30) (Fig 1F and 1G).

The *Ifne* mRNA expression pattern was validated using immunofluorescence localisation of IFNɛ protein in the 25- and 56-day-old WT mouse male reproductive tract (Fig 1H). In the male reproductive tract of WT mice at both ages, the staining was most prominent in the testis, where IFNɛ was localised to meiotic and post-meiotic spermatogenic cells, and the residual cytoplasm of spermatids within the seminiferous tubules. IFNɛ staining was very low or absent in Sertoli cells. In the interstitium, IFNɛ staining was prominent throughout the cytoplasm of testicular macrophages, with punctate staining visible in Leydig cells. IFNɛ was localised to the stereocilia in the luminal border of the epididymal caput region, especially in the initial segment of the epididymis. Staining was not observed in the corpus and cauda regions of the epididymis. The epithelium of both the proximal and distal regions of the vas deferens was positive for IFNɛ staining. IFNɛ was also localised to the efferent duct epithelium (S1A Fig). No staining was observed in *Ifne*^-/-^ mice. The gross morphology and histology of the testis and epididymis of the *Ifne* ^-/-^ mice were indistinguishable from normal (S1B and S1C Fig).

### IFNɛ is expressed in the human testis

**Fig 2:**
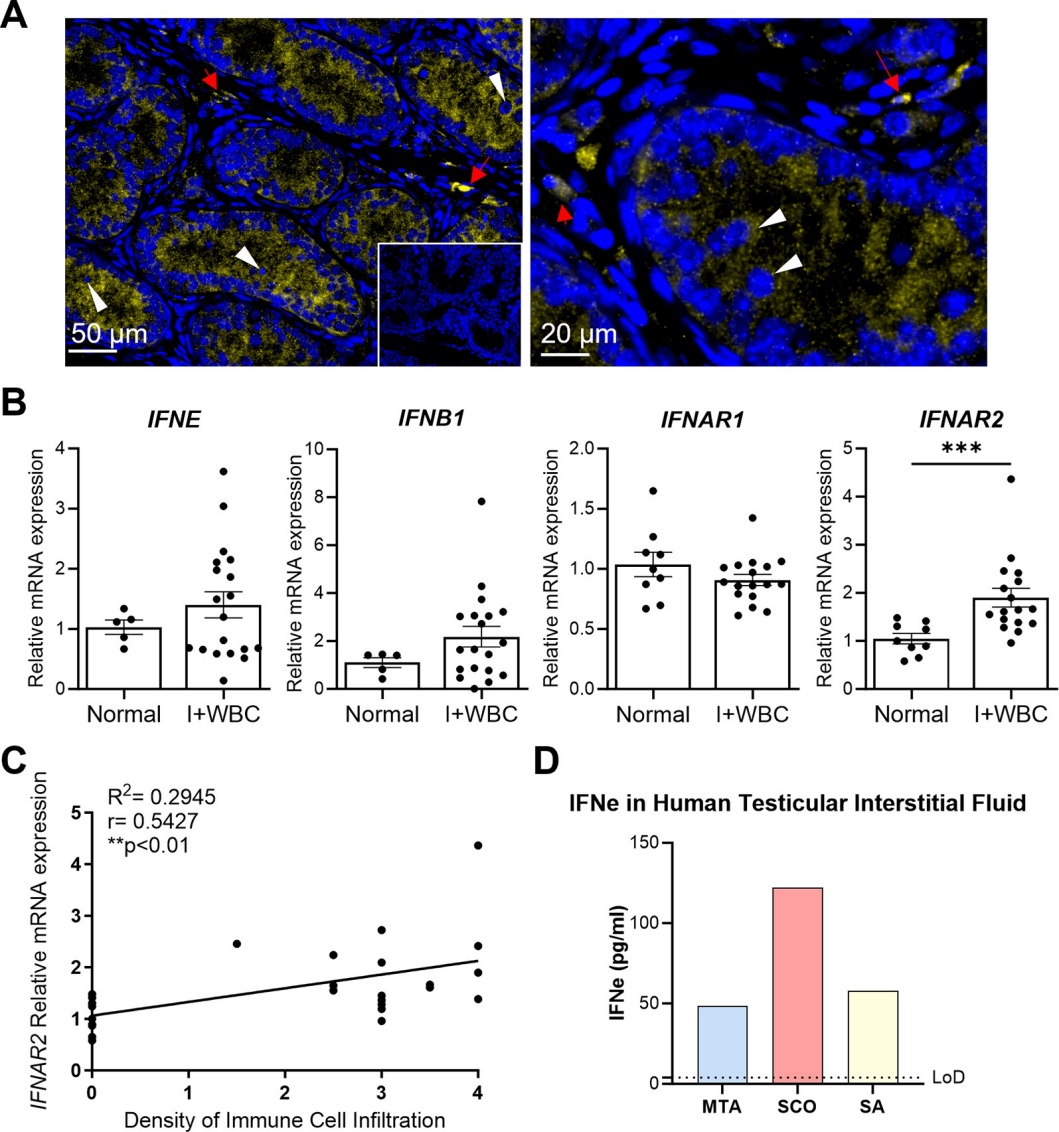
IFNɛ is constitutively expressed in the human testis. (A) IFNɛ localisation in the adult human testis, using an anti-human IFNɛ antibody at low and higher magnification; scale bars 50 and 20 μm respectively. Inset: Isotype control. Yellow: IFNɛ. Blue: DAPI. Arrows: Leydig cell and macrophage clusters. Arrowhead: post-meiotic spermatogenic cells embedded in Sertoli cell cytoplasm. Representative images from n = 3 testicular biopsy samples exhibiting normal testicular histology. (B) *IFNE*, *IFNB1* and their receptors *IFNAR1* and *IFNAR2* mRNA expression measured by qRT-PCR in testicular tissue samples showing intact spermatogenesis without any signs of inflammation (Normal) compared with biopsies showing impaired spermatogenesis and focal immune cell infiltrates (I + WBC). n = 5–19 per group. Student’s t-test, ***P < 0.001. (C) Correlation analysis of *IFNAR2* mRNA expression and the histological degree of immune cell infiltration in the testis biopsy site (scored from 0–4; 4 = dense, 3 = sparse, 2 = scattered, 1 = single cells, 0 = absent). n = 5–19 per group. Spearman’s coefficient method. (D) IFNɛ protein measured using a two-site ELISA assay in testicular interstitial fluid from three patient groups with different causes of infertility. MTA = Mixed testicular atrophy, SCO = Sertoli Cell Only phenotype, SA = spermatogenic arrest. LoD = Limit of detection: 3.9 pg/ml. Pooled samples, 25 μl testicular interstitial fluid per patient from 4 patients in each category.

Testicular biopsies with intact spermatogenesis obtained from infertile men suffering obstructive azoospermia were examined for IFNε expression. Immunofluorescence staining of these adult human testis sections showed a similar pattern to that of the mouse testis; IFNɛ was localised to the cytoplasm of meiotic and post-meiotic spermatogenic cells within the seminiferous tubules. In the interstitium, IFNɛ was localised to testicular macrophages and some Leydig cells (Fig 2A).

mRNA expression of *IFNE*, *IFNB*, and the receptors *IFNAR1* and *IFNAR2* were measured in testicular tissue specimens showing intact spermatogenesis (‘Normal’ group; median age 35 [IQR 31–40] years) and compared to biopsies from patients with impaired spermatogenesis and focal leukocyte infiltrates (‘I + WBC’ group; median age 33 [IQR 31–44] years) (Fig 2B). *IFNE* was expressed in the normal biopsies with a mean Ct value of 32, while *IFNB1* showed a mean Ct of 37. Ct values were ~25 for *IFNAR1* and ~27 for *IFNAR2* in the normal biopsies. Both *IFNE* and *IFNB1* expression showed a greater range of values in the biopsies with impaired spermatogenesis and leukocyte infiltrates, with a high variability in *IFNE* expression. *IFNAR1* expression was similar between biopsies with intact spermatogenesis and impaired spermatogenesis, but *IFNAR2* was significantly higher in the samples with impaired spermatogenesis and leukocytic infiltrates. *IFNAR2* expression showed a strong positive correlation with the density of immune cell infiltrates in the biopsies (Fig 2C), which is unsurprising since *IFNAR2* is highly expressed in immune cells. Coincidentally, *IFNE* expression in these biopsies was positively correlated with the ejaculate volume (S2A Fig) of these patients.

IFNɛ protein was measured in human testicular interstitial fluid obtained from azoospermic men with areas of intact spermatogenesis in their testicular biopsies (MTA), and compared to men with complete absence of spermatogenic cells (SCO), and no mature sperm production due to spermatogenic developmental arrest (SA), using an ELISA assay (Fig 2D). High levels of IFNɛ protein were detected in pooled samples from all three patient groups relative to the limit of detection (3.9 pg/ml), including in men with SCO phenotype. Similarly, IFNɛ protein was detected in human seminal plasma measured in pooled samples from patients with normal semen parameters (N), and patients with evidence of immunological infertility, either elevated levels of sperm auto-antibodies (Ab), or leukocytospermia (WBC) (S2B Fig).

### IFNɛ^-/-^ mice are more susceptible to epididymo-orchitis following Zika virus infection

**Fig 3:**
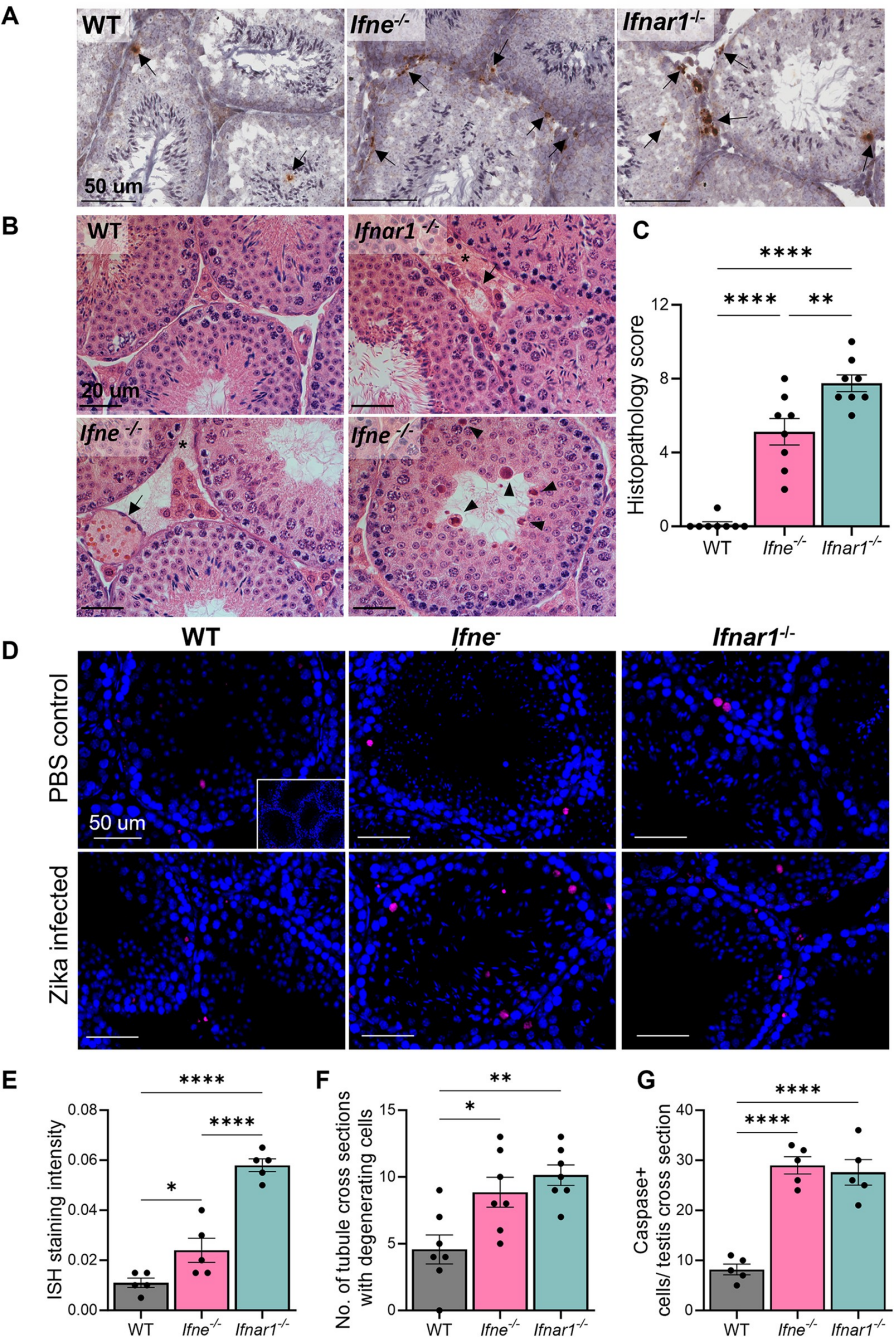
IFNɛ^-/-^ mice are more susceptible to epididymo-orchitis following Zika virus infection. (A) Detection of Zika virus RNA (arrows) by *in situ* hybridization in the testis of adult *Ifne*^-/-^ mice compared to C57BL/6 (WT) and *Ifnar1*^-/-^ mice 7 days post-Zika infection. Scale bars = 50 μm, Representative images from testis sections of 5 mice per genotype. (B) Histopathological images of Zika infected *Ifne*^-/-^ and *Ifnar1*^-/-^ mouse testes compared to infected WT mice. Vascular congestion (arrows), oedema (asterisks) and degenerating germ cells (arrowheads). PAS stain, Scale bars: 20μm, representative images from n = 8. (C) Histopathological scoring of Zika infected WT, *Ifne*^-/-^ and *Ifnar1*^-/-^ mouse testes. (D) Apoptosis detection by immunofluorescence staining for Caspase 3/9 (pink) in Zika infected testes. Blue: DAPI. Scale bars = 50 μm. Inset: negative control with scale bar of 100 μm. n = 8. (E) Quantification of ISH staining intensity presented above in Fig 3A. (F) Quantification of the number of seminiferous tubule cross sections showing at least 1 degenerating spermatogenic cell per cross section in the testes of Zika infected mice. (G) Quantification of Caspase 3/9 positive cells per testis cross section presented in Fig 3D. (For C, E-G: n = 5–8, One-Way ANOVA, *p < 0.05, **p < 0.01, ****p < 0.0001).

To determine the anti-viral role of endogenous IFNɛ expressed in the testis, the response to systemic Zika virus infection in the *Ifne*^*-/-*^ mouse male reproductive tract was compared to WT mice that do not develop clinically apparent disease [3,36]. Mice that lack the critical IFNAR1 receptor required for type I IFN signalling (*Ifnar1*^*-/-*^) were used as positive controls [36,66]. Based on testicular histopathology of *Ifne*^*-/-*^ mice at 3, 7 and 21 days post-Zika infection, the 7-day time point was selected for detailed analysis since this was when peak damage was observed (Fig 3). S3 Fig documents testicular histopathology at 3 and 21 days and testicular weights at all study timepoints.

In the testes, at 7 days post-infection, Zika viral RNA was detected in the Sertoli cells and some spermatogenic cells in the seminiferous tubules, and Leydig cells and testicular macrophages in the interstitium of *Ifne*^*-/-*^ and *Ifnar1*^*-/-*^ mice (Fig 3A). ISH staining intensity for Zika viral RNA in the WT testis was significantly lower compared to that of *Ifne*^*-/-*^ and *Ifnar1*^*-/-*^ testis section (Fig 3E). Histopathologically, both *Ifne*^*-/-*^ and *Ifnar1*^*-/-*^ mouse testes showed a significantly higher degree of inflammation-induced damage compared to WT (Fig 3B and 3C). This damage was characterized by vascular congestion and hyperemia of testicular blood vessels (arrows), oedema in the interstitial tissue (asterisk) and degenerating spermatogenic cells within the seminiferous tubules (arrowheads) (Fig 3B). *Ifne*^*-/-*^ and *Ifnar1*^*-/-*^ mouse testes contained significantly more tubule cross-sections with at least one degenerating spermatogenic cell (Fig 3F). The presence of increased numbers of apoptotic cells in both *Ifne*^*-/-*^ and *Ifnar1*^*-/-*^ mouse testes was also confirmed by immunofluorescence for caspase 3/9 (Fig 3D and 3G).

**Fig 4:**
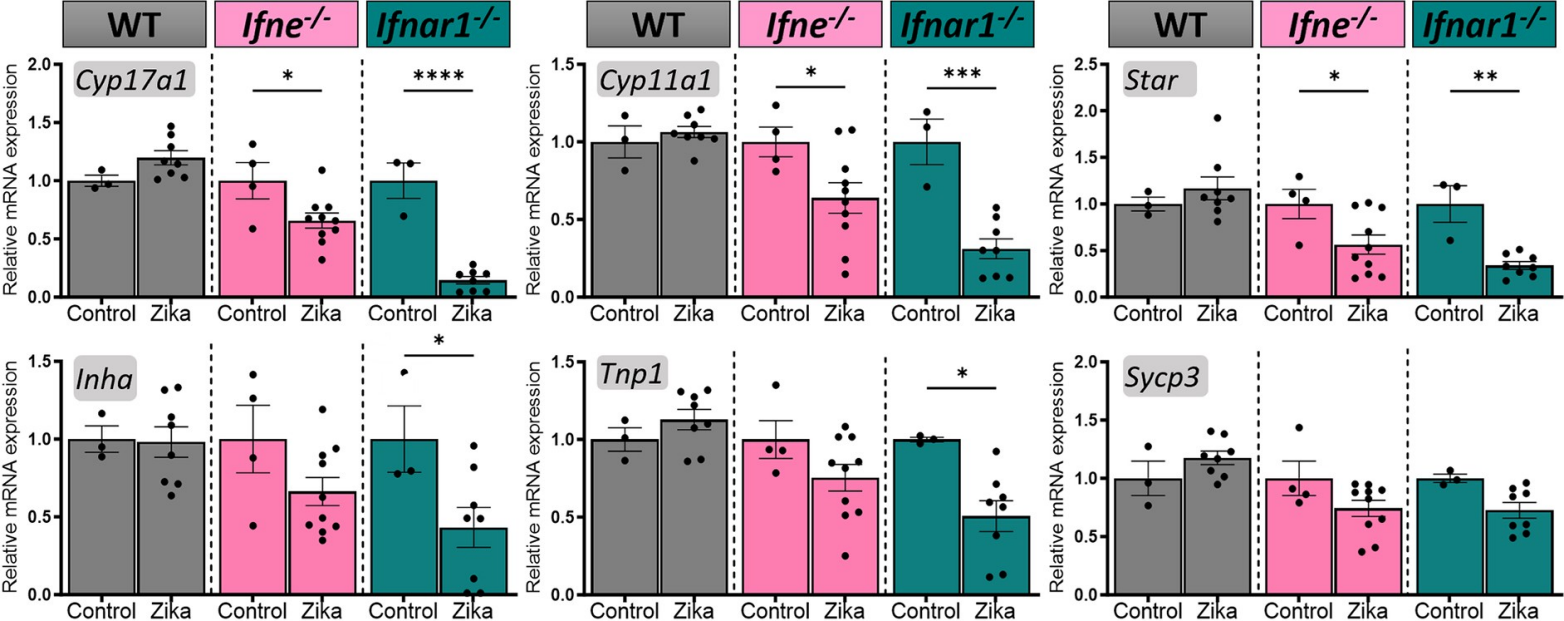
Testicular functional genes are altered in *IFNɛ*^*-/-*^ *mice following 7 day Zika virus infection*. Gene transcripts related to steroidogenesis (*Cyp11a1*, *Cyp17a1*, *Star*), Sertoli cell function (*Inha*), and spermatogenesis (*Tnp1*, *Sycp3*) measured by qRT-PCR in testes from WT (grey bars), *Ifne*^-/-^ (pink bars) and *Ifnar1*^-/-^ (green bars) mice infected with Zika virus, compared to PBS injected controls. n = 3–10 per group. 2-Way ANOVA, *p < 0.05.

Zika infection resulted in the reduction of gene transcripts involved in steroidogenesis (*Cyp17a1*, *Cyp11a1*, *Star*) measured by qRT-PCR in both *Ifne*^*-/-*^ and *Ifnar1*^*-/-*^ mice, but not in infected WT mice, and PBS injected genotype controls (Fig 4). mRNA for genes indicative of normal Sertoli cell function *(Inha)* and spermatid differentiation *(Tnp1)* were significantly reduced in the *Ifnar1*^*-/-*^ mouse testis infected with Zika, but not in the *Ifne*^*-/-*^ mice. *Sycp3*, a marker for meiosis, showed the same trend, towards reduction in most samples, but was not significantly different.

**Fig 5:**
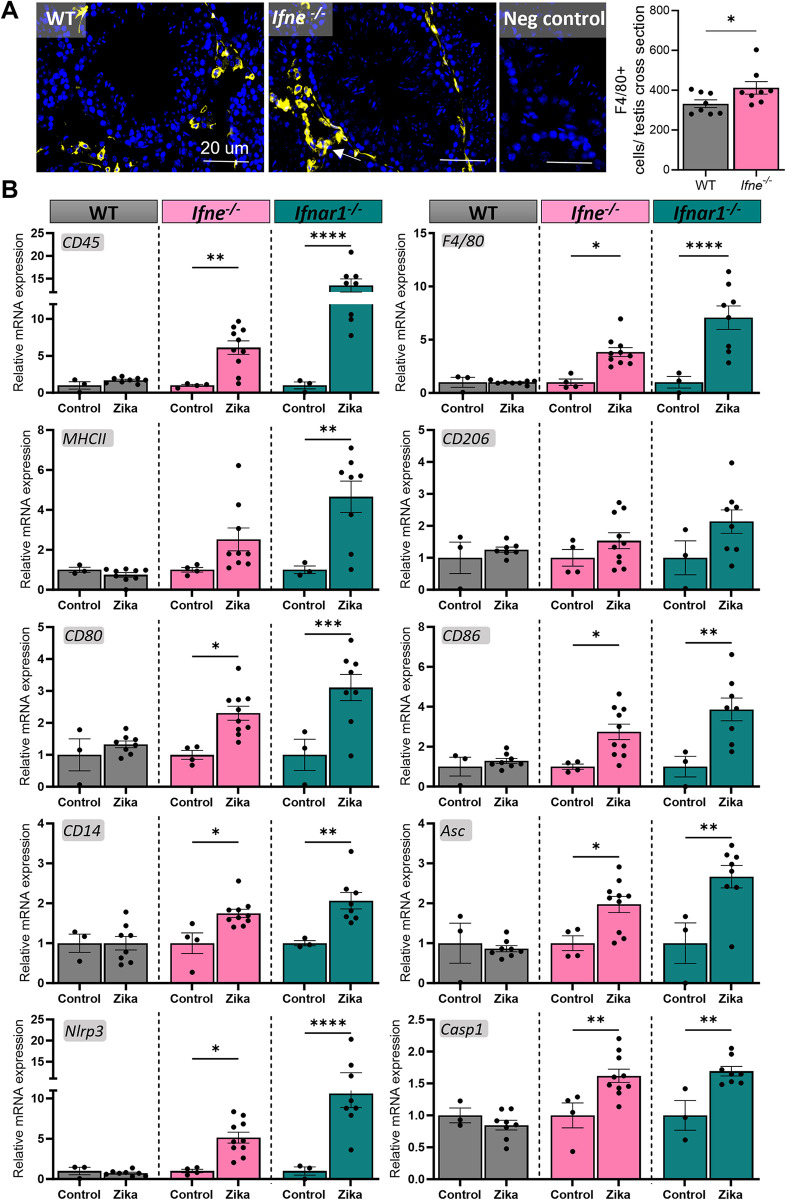
*Ifne*^-/-^ mice show testicular immune infiltrates and NLRP3 inflammasome gene upregulation 7 days post-Zika virus infection. (A) Immunofluorescence staining for F4/80+ immune cells (yellow) in the testes of Zika infected *Ifne*^-/-^ and WT mice, and graph quantifying F4/80+ cells per testis cross section. Arrow: immune cell aggregates. Blue: DAPI. Scale bar: 20 μm. (B) Gene transcripts related to immune cells and the NLRP3 inflammasome measured by qRT-PCR in testes from WT (grey bars), *Ifne*^-/-^ (pink bars) and *Ifnar1*^-/-^ (green bars) mice infected with Zika virus, compared to PBS injected controls. (n = 8–10 per group. One-Way ANOVA for graph in Fig 5A, 2-Way ANOVA for 5B, *P < 0.05, **p < 0.01, ****p < 0.0001).

Aggregates of F4/80^+^ macrophages were common in the interstitial tissue of the Zika-infected *Ifne*^*-/-*^ mice, while WT infected mice showed the normal testis macrophage distribution (Fig 5A). mRNA expression of markers of immune cells and immune cell function CD45 (*Ptprc*), F4/80 (*Adgre1*), MHC class II (I-A/I-E), CD80, CD86, and CD14 were increased in both *Ifne*^*-/-*^ and *Ifnar1*^*-/-*^ testes, with a higher degree of expression in the *Ifnar1*^*-/-*^ mice (Fig 5B). Furthermore, mRNA expression of genes involved in the NLRP3 inflammasome pathway, *Pycard* (ASC), *Nlrp3*, and *Casp1*, were increased in *Ifne*^*-/-*^ and *Ifnar1*^*-/-*^ testes, but not WT testes, 7 days post-Zika infection. The lack of inflammation or inflammatory damage in the Zika-infected WT mice occurred despite a consistent trend towards increase in splenic weight (S3C Fig), and a trend towards increase in CD3+ T cells and CD11C+ myeloid cells in the testis draining lymph nodes, indicative of peak viral infection at day 7 compared to PBS-injected WT mice (S4 and S5 Figs).

**Fig 6:**
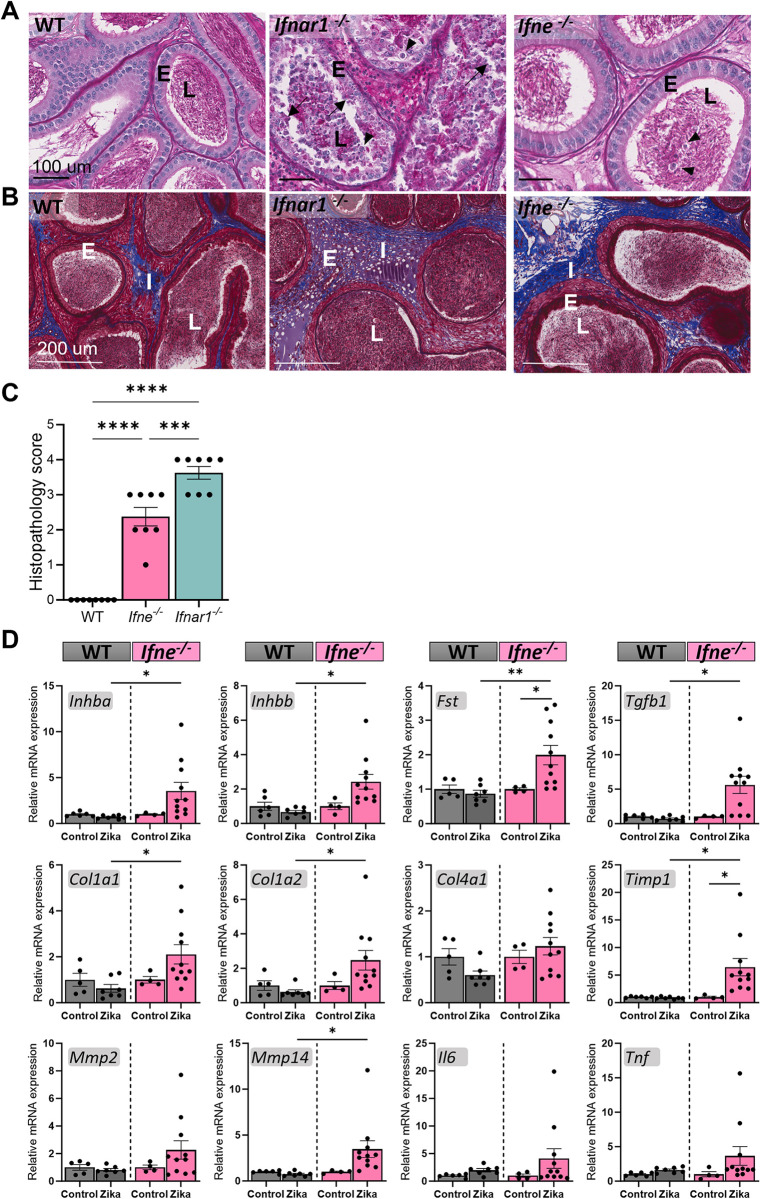
*Ifne*^-/-^ mice develop epididymitis and subsequent fibrotic damage following 7-days post-Zika virus infection. (A) Histopathological images of the epididymal corpus region of Zika infected *Ifne*^-/-^ and *Ifnar1*^-/-^ mice compared to infected WT mice. Degenerating germ cells prematurely released by the testis accumulating in the epididymal lumen (arrowheads), epithelial damage in *Ifnar1*^-/-^ mice (arrows). E: epididymal epithelium. L: lumen. I: interstitial tissue. H&E stain. Scale bars: 100 μm. (B) Histopathology to assess fibrosis in the cauda region of the epididymis of Zika infected *Ifne*^-/-^ and *Ifnar1*^-/-^ and WT mice. Masson’s trichrome stain, Scale bars: 200 μm. (C) Histopathological scoring of damage to the epididymis of Zika infected *Ifne*^-/-^ and *Ifnar1*^-/-^ mice (n = 8 per group. One-Way ANOVA, ***p < 0.001, ****p < 0.0001). (D) Gene transcripts related to fibrosis and inflammation in the cauda region of Zika infected *Ifne*^-/-^ mice compared to infected WT and uninfected *Ifne*^-/-^ and WT mice, measured by qRT-PCR. n = 6–11 per group. Kruskal-Wallis test, *p < 0.05, **p<0.01, ***p< 0.001.

The effects of systemic Zika virus infection on the epididymis, the site of sperm maturation and storage, were also examined (Fig 6). The caput region of both *Ifne*^*-/-*^ and *Ifnar1*^*-/-*^ mice did not exhibit any histopathological changes following Zika infection. The infected WT epididymis was indistinguishable from PBS-injected controls across all regions. While the epididymal corpus region of Zika-infected *Ifnar1*^*-/-*^ mice showed severe immune cell infiltration of the interstitial space and lumen, and epididymal duct epithelial disruption (arrows), the *Ifne*^*-/-*^ corpus epithelium appeared intact, and no abnormal immune cell infiltrates were detected (Fig 5A). However, degenerating spermatogenic cells (arrowheads), presumably those prematurely released from the testis, appeared in the epididymal lumen in the corpus region of *Ifne*^*-/-*^ mice, compared to infected WT mice that did not show any spermatogenic cells in the epididymal lumen. The cauda region of the *Ifne*^*-/-*^ mouse epididymis, however, showed similar levels of fibrotic damage to *Ifnar1*^*-/-*^ mice in Masson’s trichrome stained sections (Fig 6B and 6C).

Since the cauda epididymis showed the most prominent damage, a panel of inflammatory and fibrotic genes was examined in the WT and *Ifne*^*-/-*^ mouse epididymis using qRT-PCR, in Zika-infected and PBS injected control mice (Fig 6D). Hallmark genes of inflammation and fibrosis, including the activin A and B encoding genes (*Inhba*), (*Inhbb*), follistatin (*Fst*), transforming growth factor β1 (*Tgfb1*), matrix metalloproteinases including *Mmp14*, collagens (*Col1a1*, *Col1a2*) and *Timp1*, showed significant increases in the Zika infected *Ifne*^*-/-*^ mouse cauda epididymis compared to non-infected controls and the infected WT cauda. Other fibrotic genes (*Col4a1*, *Mmp2*) and the canonical inflammatory markers, *Il6* and *Tnf*, showed a variable increase that did not achieve significance.

### Zika virus infection induces most interferon stimulated genes (ISG) 48 hours post-infection in human testicular cells, while exogenous IFNɛ induces ISG production at an earlier time point

**Fig 7:**
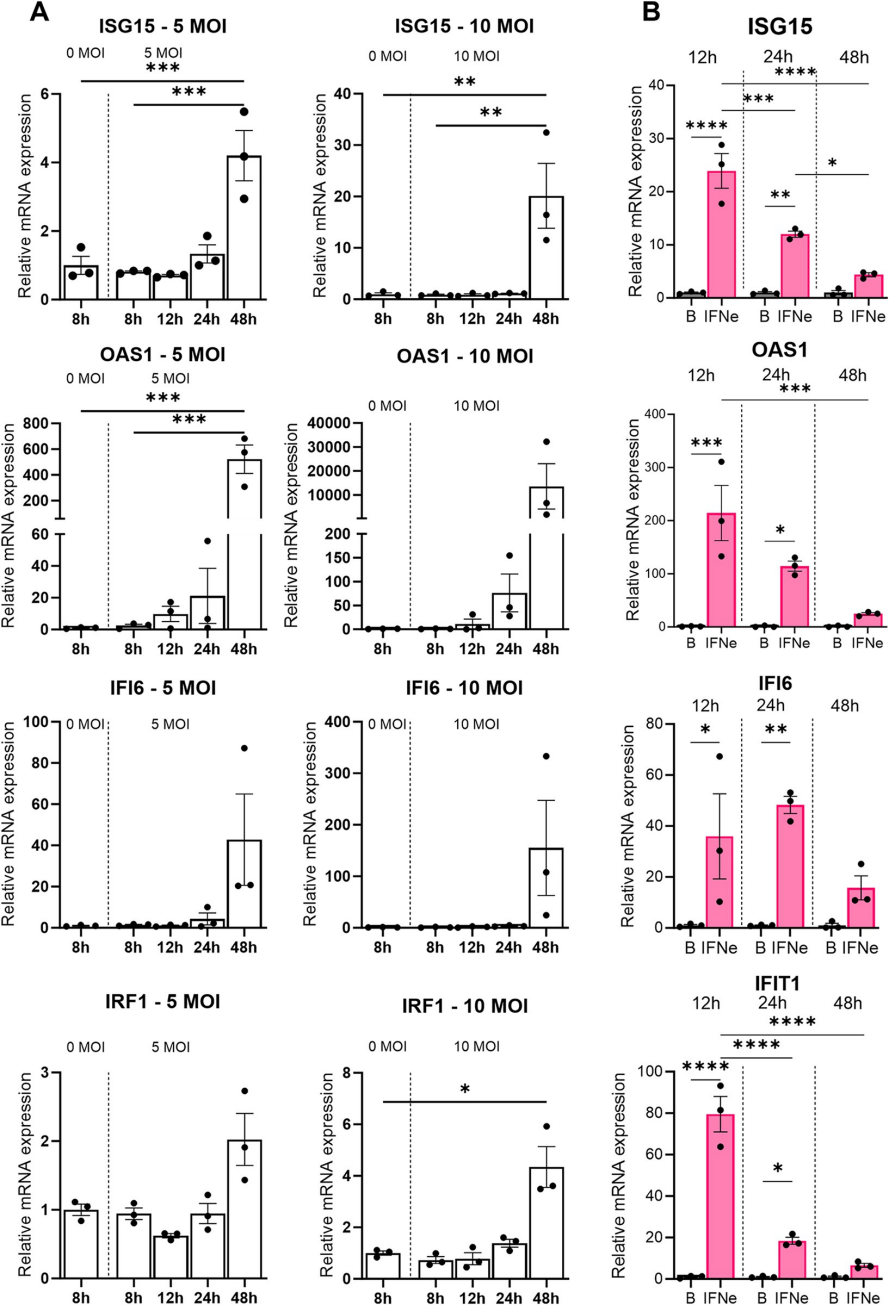
Zika virus infection induces most ISGs 48 hours post-infection in human Sertoli cells, while exogenous IFNɛ induces ISG production at an earlier time point. (A) Interferon-stimulated gene (ISG) expression in human Sertoli cell cultures measured by qRT-PCR at 8, 12, 24 and 48 hours post-infection, with either 5 or 10 MOI Zika virus, compared to uninfected controls at 8 hours. (B) ISG signature induced by 100 IU exogenous IFNɛ treatment in uninfected human Sertoli cells, measured by qRT-PCR at 12, 24 and 48 hours post-treatment, compared to buffer treated-controls. All genes were normalised to the housekeeping gene *RPLP0*, B = Buffer treated cultures, IFNɛ = IFNɛ treated cultures. Each individual data point in the graphs represent the average of three technical replicates per culture round. One-Way ANOVA to compare more than 2 data sets, Student’s t-test to compare 2 data sets, *p < 0.05, **p<0.01, ***p< 0.001, ****p< 0.0001.

Having demonstrated the crucial role IFNε plays in reducing Zika virus induced testicular damage in mice, we sought to determine the effects and mechanism of action of IFNε in the human testis. To do so, the anti-viral role of IFNɛ was examined in primary human testicular cell types and cell lines. Sertoli cells, which appear to be the principal target for type I interferons based on *Ifnar1* and *Ifnar2* receptor expression data (Fig 1E), were examined initially. Primary human Sertoli cells infected with either 5 or 10 MOI of the Zika virus showed a prominent, dose dependant, increase in the expression of ISGs including *ISG15*, *OAS1*, *IRF1*, and *IFI6* 48 hours post-infection, compared to uninfected controls at 8 hours (Fig 7A).

ISG expression was subsequently examined in uninfected primary human Sertoli cells incubated with 100 IU exogenous recombinant human IFNɛ (rhIFNɛ) for 6 hours (Fig 7B). In contrast to Zika virus infection, exogenous IFNɛ strongly induced *ISG15*, *OAS1*, *IFIT1*, and *IFI6* within 12 hours post-treatment. All other ISGs examined were significantly higher in cultures treated with IFNɛ than in controls at 24 hours post-treatment, but showed a progressive decline between 12 hours and 48 hours.

Similar to Sertoli cells, qRT-PCR assay showed that a similar dose of IFNɛ induces an equally strong ISG response in both TCam-2 cells (S6A Fig), and primary human Leydig cells (S6B Fig), 12 hours post-treatment.

### Treatment of human testicular cells with exogenous recombinant human IFNɛ has prophylactic and therapeutic effects against Zika virus infection

**Fig 8:**
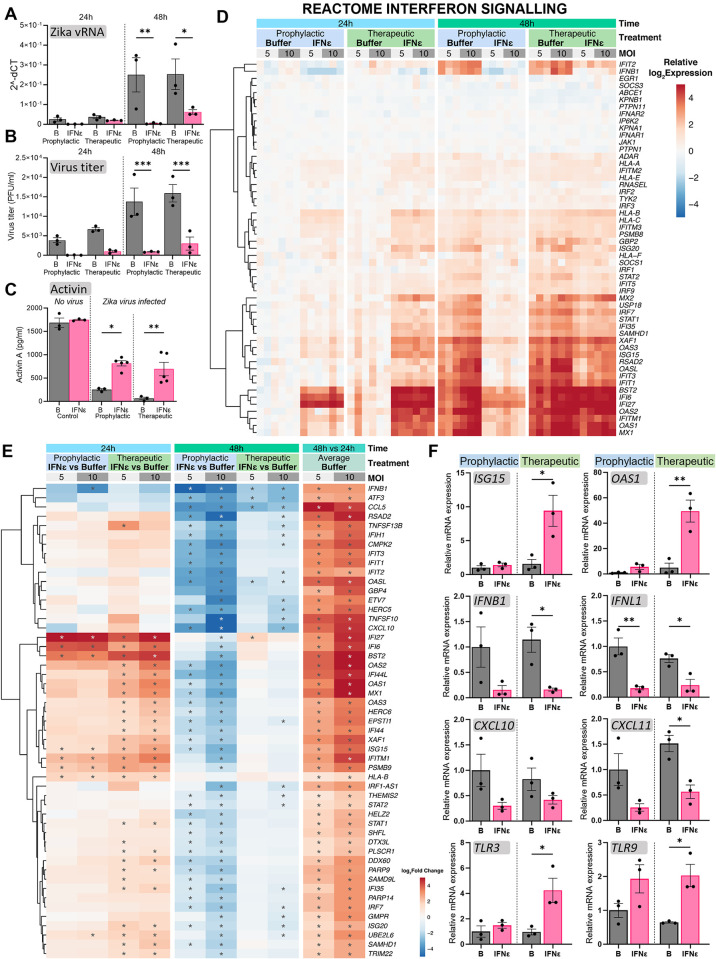
Treatment of human Sertoli cells with exogenous recombinant human IFNɛ has prophylactic and therapeutic benefits against Zika virus infection. (A) qRT-PCR detection of Zika virus RNA (2^-dCt = relative copy number) and (B) infectious viral burden measured by plaque assays in primary human Sertoli cell cultures (HSerc) treated with 100 U/ml rhIFNɛ or buffer either before (prophylactic) or after (therapeutic) infection with 5 MOI Zika virus, assayed 24 and 48 hours post-infection. (C) Activin A protein levels produced by uninfected human Sertoli cells, compared Zika infected cultures treated with either buffer or prophylactic and therapeutic IFNɛ, measured by ELISA assay of culture media. (D) RNA-seq analysis of Sertoli cell cultures treated with rhIFNɛ or buffer prophylactically or therapeutically with 5 MOI (light grey) or 10 MOI (dark grey) Zika virus, assayed 24 and 48 hours post-infection. Heat map showing the relative log_2_ gene expression (compared to the prophylactically treated buffer controls at 24 hours post-infection at 5 MOI) for genes from the Reactome interferon signalling gene set. The scale is truncated at ±5. (E) Heat map showing genes significantly differentially expressed (with a twofold treat cut-off) for any IFNɛ treatment compared to its matched buffer control. Columns show log_2_ fold changes of IFNɛ versus buffer for each condition, as well as log_2_ fold changes for 48 hours versus 24 hours with buffer alone (i.e., the effect of virus over time; prophylactic and therapeutic treatments averaged). The scale is truncated at ±5. The asterisks indicate which genes were significantly changed for which comparisons. (F) qRT-PCR of anti-viral effectors, interferons, pro-inflammatory genes, and pattern recognition receptor genes from Sertoli cell cultures infected with 5 MOI Zika virus at 24 post-infection. B = Buffer treated cultures, IFNɛ = IFNɛ treated cultures. Each individual data point in the graphs represent the average of three technical replicates per culture round. One-Way ANOVA to assess data sets with one variable, 2-Way ANOVA for 2 variables. *P < 0.05, **P < 0.01, ***P < 0.001.

To further examine the anti-viral potential of IFNɛ against Zika viral infection in the human testis, primary human Sertoli cells were treated with 100 IU rhIFNɛ either 12 hours before infection with 5 or 10 MOI Zika virus to assess a prophylactic effect or 1 hour post-Zika infection to assess a therapeutic effect. The viral load progressively increased in buffer-only cultures at 24 and 48 hours as indicated by qRT-PCR assessment of vRNA (Figs 8A and S7A) and plaque assays to measure the virus titres (Figs 8B and S7B). At both time points, prophylactic IFNε treatment reduced the viral RNA and infectious virus titres by ~98%. Therapeutic IFNɛ treatment reduced viral RNA by ~90% at 24 hours and 70% at 48 hours; infectious virus titre was reduced by 95% at 24 hours and 80% at 48 hours.

To assess if IFNɛ treatment has an effect on cell function post Zika infection, we measured the concentration of activin A, a crucial growth factor, immunoregulator, and a marker of Sertoli cell function, in the culture media. Zika virus significantly reduced Sertoli cell secretion of activin A (Fig 8C), while both prophylactic and therapeutic IFNɛ treatments significantly increased activin A secretion compared to controls.

To assess if IFNε was effective against virus infection in other human testicular cell types, infected TCam-2 cells and primary human Leydig cells treated with or without IFNε were assessed 24 hours post-infection. Similar to that observed for Sertoli cells, prophylactic and therapeutic treatment with IFNε significantly reduced vRNA and the infectious virus titre in TCam-2 and Leydig cells (S8A and S8B Fig). While peak viral titres in buffer-only treated Sertoli cells reached 2 × 10^5^ PFU/ml at 48 hours post-infection (Fig 8B), Zika viral titres were considerably lower at this time point in TCam-2 cells and Leydig cells (peak of ~500 PFU/ml).

### Exogenous IFNε treatment confers anti-viral protection in human Sertoli cells by inducing a strong interferon response at 24 hours

To understand the mechanisms by which IFNε confers anti-viral protection to testicular cells, RNA sequencing was performed on the Zika-infected human Sertoli cells treated prophylactically or therapeutically with 100 IU rhIFNε. Both prophylactic and therapeutic IFNε treatment induced a strong interferon-driven anti-viral response 24 hours post-infection (Figs 8D, S7C and S7D). Classic ISGs, including *ISG15*, *IFI6*, and *IFITM1* were significantly upregulated following both prophylactic and therapeutic IFNε treatment. *OAS1* was increased with therapeutic treatment (Figs 8D and 7E and S4 Table). *IFNα* genes were not detected at either time point examined. *IFNB1* and *IFNL1* were reduced following IFNε treatment, but were strongly induced by Zika virus in buffer treated control cultures (Fig 8E and 8F and S4 Table).

At 48 hours post-infection, however, a relative suppression of interferon gene sets and anti-viral effector gene sets occurred in the IFNε-treated groups, compared to buffer-treated cultures, particularly with the prophylactic treatment (Figs 8D, 8E and S9), which can be attributed to the reduced viral load at this time-point due to the action of IFNε (Fig 8A and 8B).

Inflammatory gene sets, and individual inflammatory maker genes *CXCL10*, *CXCL11* and *CCL5*, were significantly increased in the buffer-only treated cultures from 24 hours to 48 hours, in a viral dose-dependent manner, indicating that the virus infection drives this expression over time (Fig 8F and S7C). Both prophylactic and therapeutic IFNε treatment reduced the expression of inflammatory marker gene sets at 24 hours post-infection, although this reduction did not achieve statistical significance. At 48 hours post-infection, however, inflammatory marker gene sets were significantly reduced. *CXCL10* and *CXCL11* gene data were validated using RT-qPCR (Figs 8F and S9). Furthermore, *TLR3* and *TLR9*, pattern recognition receptor genes specific for viral detection, were increased following IFNε treatment (Figs 8F and S4).

Finally, to assess if exogenous IFNε treatment conferred anti-viral protection to other testicular cells similar to human Sertoli cells, we examined a selected panel of genes in TCam-2 and Leydig cells 24 hours post-Zika infection (Fig S8C and S8D). Anti-viral effectors *ISG15* and *OAS1* were increased in both cell types following exogenous IFNε treatment, with therapeutic treatment resulting in a greater increase, compared to prophylactic treatment, similar to the pattern observed in Sertoli cells. Both prophylactic and therapeutic treatment reduced *CXCL10* and *CXCL11* in TCam-2 and Leydig cells. *IFNB1* was reduced, compared to buffer only treated controls. *TLR3* was increased in both cell types following IFNε treatment.

## Discussion

Testicular viral infections have adverse impacts on fertility, testosterone levels, and overall health of the individual, while carrying the risk of disease transmission to partners and offspring. The elucidation of the anti-viral defences of the testis is crucial to understanding how viruses infect the testis and why they can persist, if we are to discover rational treatments to prevent or eliminate these infections and their subsequent threats to reproductive health. This is the first study to closely examine the production of IFNε in the testis and demonstrates that constitutive IFNε plays a critical role in antiviral defence in both the murine and human male reproductive tract. This is especially important because spermatogenic cells were thought to be devoid of self-protective anti-viral mechanisms, since they do not express classical inducible type I interferons such as IFNβ and ISGs [11,12]. Thus, the constitutive expression of IFNε in meiotic and post-meiotic spermatogonial cells indicate a crucial role for this non-classical type I interferon, to compensate for the deficiencies in the inducible anti-viral responses in the testis.

IFNε mRNA first appears in the murine testis between day 20–25, coinciding with the completion of the first wave of meiosis and appearance of haploid spermatogenic cells. In the female reproductive tract, IFNε appears to be expressed entirely by the epithelium [18,19]. In the male, however, IFNε is expressed by the meiotic and post-meiotic spermatogonial cells in the seminiferous epithelium, as well as by the testicular macrophages and testosterone-producing Leydig cells in the interstitium. Sertoli cells, an epithelial cell type that provides nutrition and protection to developing spermatogenic cells, on the other hand, appear to express relatively low levels of IFNε in the mouse. Men with the SCO phenotype that lack any spermatogenic cells express high levels of IFNε mRNA and protein, highlighting the contribution of non-spermatogenic cells to the levels of IFNε in the testis. While it would appear that sexual maturation drives the expression of IFNε, the relative contributions of the spermatogenic cells and somatic cells to IFNε levels in the testis compartments requires further examination. The window when IFNε first appears in the testis also coincides with rising testosterone levels. In the female, IFNε is dependent upon oestrogen and progesterone. Given the differences in IFNε expression sites between the male and female reproductive tracts, hormonal regulation of IFNε in the male is an important area that still needs to be examined.

To assess the role of endogenous IFNε in testicular antiviral defence in mice, we used a murine model of systemic Zika virus infection. Histologically, at 7 days post-infection, the *Ifne*^*-/-*^ mouse testis showed signs of inflammatory damage comparable to that seen in the *Ifnar1*^*-/-*^ mouse, which is unable to mount a type I interferon response entirely. In addition to vascular congestion and oedema in the interstitium, the Zika virus induced apoptosis of spermatogenic cells in the *Ifne*^*-/-*^ and *Ifnar1*^*-/-*^ mouse testis. Sertoli cells are known to produce CXCL10 in response to viral infection, which triggers cell apoptosis of spermatogenic cells through activation of caspase 3 [67], and their premature displacement from the seminiferous epithelium. These spermatogenic cells were then observed in the epididymis, the site where sperm undergo maturation and storage. While IFNε was not expressed in the epididymis, *Ifne*^*-/-*^ mice developed fibrotic damage in the cauda region of the epididymis. Fibrotic obstruction of the cauda epididymis is known to adversely affect male fertility [68]. Autoantigenic spermatogenic cells that are prematurely released from the testis during infection are known to trigger fibrosis in the epididymis, which unlike the testis does not have an immune-privileged environment [69]. The corpus region of the *Ifnar1*^*-/-*^ mice infected with Zika showed substantial damage compared to *Ifne*^*-/-*^ mice, potentially because the of lack of other type I interferons that might be crucial in the epididymis.

At the mRNA level, infected *Ifne*^*-/-*^ mice had reduced levels of key steroidogenic genes and genes associated with spermatogenesis, indicating damage to testis function. However, this reduction was intermediate in severity between WT mice that did not show any signs of damage, and *Ifnar1*^*-/-*^ mice that had drastically reduced gene transcripts. These data demonstrate that endogenous IFNε plays a critical role in testicular anti-viral defence against the Zika virus in conjunction with other inducible type I interferons, similar to that observed in the female reproductive tract [17].

Previous studies show that Sertoli cells are a key target of the Zika virus, leading to damage of the blood-testis-barrier composed by the tight junctions between Sertoli cells, and subsequent disruption of spermatogenesis [2,3,40]. The Zika virus induced a strong ISG response at 48 hours post-infection in the present study. However, stimulation of Sertoli cells with exogenous IFNε in the absence of infection produced an earlier potent ISG response at 12 hours post-treatment. This suggests that constitutive expression of IFNε in the testis provides crucial early defence against the virus, before Sertoli cells can mount an anti-viral response. Treatment of Zika virus-infected human Sertoli cells with exogenous IFNε had prophylactic and therapeutic effects in significantly reducing the infectious viral load and improving activin A secretion by infected Sertoli cells. Prophylactic and therapeutic exogenous IFNε also reduced the infectious viral burden in TCam-2 and Leydig cells. In all cell types, prophylactic treatment was more effective in reducing the virus RNA and infectious virus titer, compared to therapeutic IFNε, reaching a decrease of up to ~98% in some cases. This provides further evidence that constitutive expression of IFNε expression is important for early virus detection and elimination in the testis.

To identify the mechanisms by which IFNε confers anti-viral protection in the testis, we performed RNA sequencing of Zika-infected human Sertoli cells treated with exogenous IFNε either prophylactically or therapeutically. The antiviral response generated by IFNε in Sertoli cells was characterized by a typical interferon antiviral signature. A study that used the same dose of exogenous IFNε on human female reproductive tract cell lines showed that IFNε, IFNα and IFNλ all induced a similar gene signature, but the IFNε-driven response was earlier than that of IFNλ, and less potent than the response by IFNα [17].

Similar to this observation in female reproductive tract cells [17], IFNε induced several ISGs known to inhibit Zika viral infection in Sertoli cells, TCam-2 cells and Leydig cells. Interestingly, the magnitude of induction of *IRF1* was relatively low compared to other ISGs induced by these cell types, as for exogenous IFNε-stimulated female reproductive cells [17]. Pattern recognition receptors involved in detecting viruses *TLR3* and *TLR9* were upregulated in all three types of testicular cells in response to IFNε treatment. This indicates that constitutive IFNε may play a role in improving viral detection and inducing an early anti-viral response in the testis. Pro-inflammatory cytokines were reduced following IFNε treatment, potentially due to the reduced viral load in these cells.

Of the three types of testicular cells used in the *in vitro* experiments, Sertoli cells showed the highest Zika viral burden. In mice, Sertoli cells show low levels of endogenous IFNε, but express very high levels of *Ifnar1* and *Ifnar2* receptor mRNA. Meiotic and post-meiotic spermatogenic cells that express high levels of endogenous IFNε show low levels of *Ifnar1* and *Ifnar2* receptor mRNA expression. Taken together, it appears that Sertoli cells are probably the principal target for the endogenous IFNε produced in the testis, inducing early anti-viral responses in this cell type that play a key role in testicular defence.

In conclusion, this study shows that constitutive IFNε expression in the testis is a crucial antiviral defence mechanism in the male reproductive tract and its absence leads to inflammation induced damage to male fertility following Zika virus infection. Clearly, it will be important to assess whether differences in IFNε regulation or activity contributes to the susceptibility to viral infection, persistence and severity of orchitis in human patients.

## Supporting information

S1 Fig IFNɛ constitutive expression in the reproductive tract of male mice and characterization of the uninfected *Ifne*^-/-^ mouse testis and epididymis. (A) Immunofluorescence staining for IFNɛ in the distal region of the WT mouse vas deferens and efferent ducts at 56 and 25 days of age. Scale bars = 50 μm. (B) Testicular and epididymal weights at 25 and 56 days of age in *Ifne*^-/-^ mice compared to WT controls. (C) Testicular and epididymal histology of *Ifne*^-/-^ mice compared to WT controls at 56 days of age. Representative images from n = 4 mice per genotype. Scale bars = 100μm.
(TIF)

S2 Fig IFNɛ in the human testis. (A) Correlation between IFNɛ mRNA measured by qRT-PCR in testicular tissue samples and ejaculation volume in a cohort of infertile men. (B) IFNɛ protein measured using a two-site ELISA assay in human seminal plasma from normozoospermic men (N), patients with antibodies (Ab) and patients with leukocytospermia (WBC) (Limit of detection/Assay sensitivity: 3.9 pg/ml. Pooled samples, 25μl testicular interstitial fluid per patient from 4 patients in each category).
(TIF)

S3 Fig Testicular histology 3 and 21 days post-Zika infection in *Ifne*^-/-^ and *Ifnar1*^-/-^ mice compared to WT mice. (A) Vascular congestion (arrows), oedema (asterisk) and degenerating germ cells (arrowheads). (B) Combined weights of right and left testes (in mg) 3, 7 and 21 days post-Zika infection. (C) Spleen weight (mg) 3, 7 and 21 days post-Zika infection. *Ifnar1*^-/-^ mice were only examined at 3 and 7 days due to ethical limitations. Representative images from n = 3 mice per experimental group. Scale bars = 100μm.
(TIF)

S4 Fig Flow cytometry quantification of T cells in mouse testes-draining lymph nodes after ZIKV infection. CD3 T cells counts in PBS injected WT controls, Zika infected WT, PBS injected *Ifne*^-/-^ controls and Zika infected *Ifne*^-/-^ mice Data are presented as mean ± SD.
(TIF)

S5 Fig Flow cytometry quantification of myeloid cell counts in mouse testes-draining lymph nodes after ZIKV infection. (A) CD11c+ and (B) CD11c+CD11b+ dendritic cell (DC) counts. (C) CD11b+ and (D) CD11b+CD11c+ Macrophage (MPh) counts. MPh were defined as F4/80+CD11b+CD11c±. Data are presented as mean ± SD.
(TIF)

S6 Fig Exogenous IFNɛ induces ISG production at 12 hours in human seminoma cells and primary Leydig cells. ISGs induced 12 hours post-treatment by 100 IU exogenous IFNɛ, in an uninfected (A) human seminoma cell line (TCam-2) and (B) human Leydig cells. All genes were normalised to the housekeeping gene *RPLP0*, B = Buffer treated cultures, IFNɛ = IFNɛ treated cultures. Each individual data point in the graphs represent the average of three technical replicates per culture round. One-Way ANOVA to compare more than 2 data sets, Student’s t-test to compare 2 data sets, *p < 0.05, **p<0.01, ***p< 0.001.
(TIF)

S7 Fig Treatment of human Sertoli cells with exogenous recombinant human IFNɛ has prophylactic and therapeutic benefits against Zika virus infection at 10 MOI dose. (A) qRT-PCR detection of Zika virus RNA in primary human Sertoli cell cultures (HSerc) treated with 100 U/ml rhIFNɛ or buffer either before or after infection with 10 MOI Zika virus, assayed 24 and 48 hours post-infection (2^-dCt = relative copy number) (B) Infectious viral burden in Sertoli cell culture media measured by plaque assays 24 and 48 hours post-infection with 10 MOI Zika virus B = Buffer treated cultures, IFNɛ = IFNɛ treated cultures. Each individual data point in the graphs represent the average of three technical replicates per culture round. 2-Way ANOVA, *P < 0.05, **P < 0.01***P < 0.001. (C–D) Heat maps showing some of the most significant gene sets from the MsigDB Hallmark (C) and Reactome (D) gene set collections, associated with the RNA-seq analysis of human Sertoli cells, with each rhIFNɛ treatment compared to its matched buffer control, assessed using the cameraPR function. The colours indicate the average log_2_ fold changes of all genes in the gene set for each comparison, with the scale truncated to ±2. The significance of each gene set is Indicated by the text as the −log_10_ FDR-adjusted p-value. * denotes adjusted p-value threshold p < 0.05, 2 denotes p < 0.01, 3 denotes p < 0.001 and so on.
(TIF)

S8 Fig Exogenous IFNɛ treatment confers anti-viral protection in human Leydig cells and a seminoma cell line (TCam-2) by inducing a strong interferon response at 24 hours. qRT-PCR detection of Zika virus RNA, and infectious viral burden measured by plaque assays in (A) a human Seminoma cell line, and (B) primary human Leydig cell cultures (HLC) treated with 100 U/ml rhIFNɛ or buffer either before (prophylactic) or after (therapeutic) infection with 5 MOI Zika virus, assayed 24 hours post-infection (2^-dCt = relative copy number). (C) qRT-PCR of anti-viral effectors, interferons, pro-inflammatory genes, and pattern recognition receptor genes from TCam-2 cells and (D) primary human Leydig cell cultures infected with 5 MOI Zika virus at 24 hours post-infection. B = Buffer treated cultures, IFNɛ = IFNɛ treated cultures. Each individual data point in the graphs represent the average of three technical replicates per culture round. One-Way ANOVA to assess data sets with one variable, 2-Way ANOVA for 2 variables. *P < 0.05, **P < 0.01, ***P < 0.001, ****P < 0.0001.
(TIF)

S9 Fig qRT-PCR validation of selected anti-viral and pro-inflammatory genes from Sertoli cell cultures infected with 10 MOI Zika virus at 24 and 48 hours post-infection. B = Buffer treated cultures, IFNɛ = IFNɛ treated cultures. Each individual data point in the graphs represent the average of three technical replicates per culture round. 2-way ANOVA, *P < 0.05.)
(TIF)

S1 Table qRT-PCR primers used in the study. (PDF)

S2 Table Histopathological damage scoring in mouse testis sections. (PDF)

S3 Table Histopathological damage scoring in mouse epididymis sections. (PDF)

S4 Table Excel spreadsheet containing additional RNA-seq data too large to fit in a PDF. (XLSX)

## References

[1] Le Tortorec A, Matusali G, Mahé D, Aubry F, Mazaud-Guittot S, Houzet L, Dejucq-Rainsford N. From Ancient to Emerging Infections: The Odyssey of Viruses in the Male Genital Tract. Physiological reviews (2020). 100, 1349–1414. doi: 10.1152/physrev.00021.2019 32031468

[2] Tsetsarkin KA, Acklin JA, Liu G, Kenney H, Teterina NL, Pletnev AG, Lim JK. Zika virus tropism during early infection of the testicular interstitium and its role in viral pathogenesis in the testes. PLOS Pathogens (2020). 16, e1008601. 32614902 10.1371/journal.ppat.1008601 PMC7331987

[3] Govero J, Esakky P, Scheaffer SM, Fernandez E, Drury A, Platt DJ, Gorman MJ, Richner JM, Caine EA, Salazar V., et al. Zika virus infection damages the testes in mice. Nature (2016). 540, 438–442. doi: 10.1038/nature20556 27798603 PMC5432198

[4] Duarte-Neto AN, Teixeira TA, Caldini EG, Kanamura CT, Gomes-Gouvêa MS, Dos Santos ABG, Monteiro RAA, Pinho JRR, Mauad T, da Silva LFF, et al. Testicular pathology in fatal COVID-19: A descriptive autopsy study. Andrology (2022). 10, 13–23. doi: 10.1111/andr.13073 34196475 PMC8444746

[5] Pilatz A, Arneth B, Kaiser R, Heger E, Pirkl M, Böttcher S, Fritzenwanker M, Renz H, Mankertz A, Schuppe HC, Wagenlehner F. Acute orchitis deciphered: Coxsackievirus B strains are the main etiology and their presence in semen is associated with acute inflammation and risk of persistent oligozoospermia. J Med Virol (2023). 95, e28970. doi: 10.1002/jmv.28970 37477797

[6] Hedger MP. Immunophysiology and Pathology of Inflammation in the Testis and Epididymis. Journal of Andrology (2011). 32, 625–640. doi: 10.2164/jandrol.111.012989 21764900 PMC7166903

[7] Washburn RL, Hibler T, Kaur G, Dufour JM. Sertoli Cell Immune Regulation: A Double-Edged Sword. Front Immunol (2022). 13, 913502. doi: 10.3389/fimmu.2022.913502 35757731 PMC9218077

[8] Doyle TJ, Kaur G, Putrevu SM, Dyson EL, Dyson M, McCunniff WT, Pasham MR, Kim KH, Dufour JM. Immunoprotective properties of primary Sertoli cells in mice: potential functional pathways that confer immune privilege. Biol Reprod (2012). 86, 1–14. doi: 10.1095/biolreprod.110.089425 21900683 PMC3313662

[9] Meinhardt A, Hedger MP. Immunological, paracrine and endocrine aspects of testicular immune privilege. Molecular and Cellular Endocrinology (2011). 335, 60–68. doi: 10.1016/j.mce.2010.03.022 20363290

[10] Kuassivi ON, Abiven H, Satie AP, Cartron M, Mahé D, Aubry F, Mathieu R, Rebours V, Le Tortorec A, Dejucq-Rainsford N. Human Testicular Germ Cells, a Reservoir for Zika Virus, Lack Antiviral Response Upon Zika or Poly(I:C) Exposure. Front Immunol (2022). 13, 909341. doi: 10.3389/fimmu.2022.909341 35784373 PMC9248283

[11] Dejucq N, Lienard MO, Guillaume E, Dorval I, Jégou B. Expression of interferons-alpha and -gamma in testicular interstitial tissue and spermatogonia of the rat. Endocrinology (1998). 139, 3081–3087. doi: 10.1210/endo.139.7.6083 9645679

[12] Dejucq N, Liénard MO, Jégou B. Interferons and interferon-induced antiviral proteins in the testis. J Reprod Immunol (1998). 41, 291–300. doi: 10.1016/s0165-0378(98)00065-5 10213317

[13] Purpura LJ, Choi MJ, Rollin PE Zika virus in semen: lessons from Ebola. The Lancet Infectious Diseases (2016). 16, 1107–1108. 10.1016/S1473-3099(16)30330-9 27676341

[14] Coffin KM, Liu J, Warren TK, Blancett CD, Kuehl KA, Nichols DK, Bearss JJ, Schellhase CW, Retterer CJ, Weidner JM, et al. Persistent Marburg Virus Infection in the Testes of Nonhuman Primate Survivors. Cell host & microbe (2018). 24, 405–416.e403. doi: 10.1016/j.chom.2018.08.003 30173956

[15] Dejucq N, Jégou B. Viruses in the Mammalian Male Genital Tract and Their Effects on the Reproductive System. Microbiology and Molecular Biology Reviews (2001). 65, 208–231. doi: 10.1128/MMBR.65.2.208-231.2001 11381100 PMC99025

[16] Teixeira TA, Oliveira YC, Bernardes FS, Kallas EG, Duarte-Neto AN, Esteves SC, Drevet JR, Hallak J. Viral infections and implications for male reproductive health. Asian journal of andrology (2021). 23, 335–347. doi: 10.4103/aja.aja_82_20 33473014 PMC8269834

[17] Coldbeck-Shackley RC, Romeo O, Rosli S, Gearing LJ, Gould JA, Lim SS, Van der Hoek KH, Eyre NS, Shue B, Robertson SA, et al. Constitutive expression and distinct properties of IFN-epsilon protect the female reproductive tract from Zika virus infection. PLoS Pathog (2023). 19, e1010843. doi: 10.1371/journal.ppat.1010843 36897927 PMC10032502

[18] Fung KY, Mangan NE, Cumming H, Horvat JC, Mayall JR, Stifter SA, De Weerd N, Roisman LC, Rossjohn J, Robertson SA, et al. Interferon-ε protects the female reproductive tract from viral and bacterial infection. Science (2013). 339, 1088–1092. 23449591 10.1126/science.1233321 PMC3617553

[19] Marks ZRC, Campbell N, deWeerd NA, Lim SS, Gearing LJ, Bourke NM, Hertzog PJ. Properties and functions of the novel type I Interferon Epsilon. Seminars in immunology (2019). 43, 101328. doi: 10.1016/j.smim.2019.101328 31734130

[20] Marks ZRC, Campbell NK, Mangan NE, Vandenberg CJ, Gearing LJ, Matthews AY, Gould JA, Tate MD, Wray-McCann G, Ying L, et al. Interferon-ε is a tumour suppressor and restricts ovarian cancer. Nature (2023). 620, 1063–1070. 37587335 10.1038/s41586-023-06421-w

[21] Hermant P, Francius C, Clotman F, Michiels T. IFN-ε is constitutively expressed by cells of the reproductive tract and is inefficiently secreted by fibroblasts and cell lines. PLoS One (2013). 8, e71320. 23951133 10.1371/journal.pone.0071320 PMC3739789

[22] D’Ortenzio E, Matheron S, Yazdanpanah Y, de Lamballerie X, Hubert B, Piorkowski G, Maquart M, Descamps D, Damond F, Leparc-Goffart I. Evidence of Sexual Transmission of Zika Virus. The New England journal of medicine (2016). 374, 2195–2198. doi: 10.1056/NEJMc1604449 27074370

[23] Duggal NK, McDonald EM, Ritter JM, Brault AC. Sexual transmission of Zika virus enhances in utero transmission in a mouse model. Sci Rep (2018). 8, 4510. doi: 10.1038/s41598-018-22840-6 29540804 PMC5852059

[24] Epelboin S, Dulioust E, Epelboin L, Benachi A, Merlet F, Patrat C. Zika virus and reproduction: facts, questions and current management. Hum Reprod Update (2017). 23, 629–645. doi: 10.1093/humupd/dmx024 28961800

[25] Fietz D, Kliesch S. Biopsy and Histology of the Testis. In Andrology: Male Reproductive Health and Dysfunction, E. Nieschlag HM. Behre S, Kliesch S Nieschlag, eds. (Springer International Publishing), pp. (2023). 181–196.

[26] O’Donnell L, Dagley LF, Curley M, Darbey A, O’Shaughnessy PJ, Diemer T, Pilatz A, Fietz D, Stanton PG, Smith LB, Rebourcet D. Sertoli cell-enriched proteins in mouse and human testicular interstitial fluid. PLoS One (2023). 18, e0290846. doi: 10.1371/journal.pone.0290846 37656709 PMC10473511

[27] World Health Organization. (2010). WHO laboratory manual for the examination and processing of human semen. 5th ed ed. World Health Organization.

[28] World Health Organization. (2021). WHO laboratory manual for the examination and processing of human semen, 6th ed Edition (World Health Organization).

[29] Hasan H, Peng W, Wijayarathna R, Wahle E, Fietz D, Bhushan S, Pleuger C, Planinić A, Günther S, Loveland KL, et al. Monocytes expressing activin A and CCR2 exacerbate chronic testicular inflammation by promoting immune cell infiltration. Human reproduction (Oxford, England). (2024). doi: 10.1093/humrep/deae107 38775335

[30] Ochsenkühn R, O’Connor AE, Hirst JJ, Gordon Baker HW, de Kretser DM, Hedger MP. The relationship between immunosuppressive activity and immunoregulatory cytokines in seminal plasma: influence of sperm autoimmunity and seminal leukocytes. J Reprod Immunol (2006). 71, 57–74. doi: 10.1016/j.jri.2006.01.002 16712948

[31] Hedger MP, Eddy EM The Heterogeneity of Isolated Adult Rat Leydig Cells Separated on Percoll Density Gradients: An Immunological, Cytochemical, and Functional Analysis. Endocrinology (1987). 121, 1824–1838. doi: 10.1210/endo-121-5-1824 2822376

[32] Scarpino S, Rita Morena A, Petersen C, Fröysa B, Söder O, Boitani C. A rapid method of Sertoli cell isolation by DSA lectin, allowing mitotic analyses. Molecular and Cellular Endocrinology (1998). 146, 121–127. doi: 10.1016/s0303-7207(98)00190-7 10022769

[33] Winnall WR, Okuma Y, Saito K, Muir JA, Hedger MP. Regulation of interleukin 1α, activin and inhibin by lipopolysaccharide in Sertoli cells from prepubertal rats. Molecular and Cellular Endocrinology (2009). 307, 169–175. 19524137 10.1016/j.mce.2009.02.007

[34] Sluka P, O’Donnell L, Bartles JR, Stanton PG. FSH regulates the formation of adherens junctions and ectoplasmic specialisations between rat Sertoli cells in vitro and in vivo. Journal of Endocrinology (2006). 189, 381–395. doi: 10.1677/joe.1.06634 16648304

[35] Loveland KL, Hedger MP, Risbridger G, Herszfeld D, De Kretser DM. Identification of receptor tyrosine kinases in the rat testis. Molecular reproduction and development (1993). 36, 440–447. doi: 10.1002/mrd.1080360406 8305206

[36] Lazear HM, Govero J, Smith AM, Platt DJ, Fernandez E, Miner JJ, Diamond MS. A Mouse Model of Zika Virus Pathogenesis. Cell host & microbe (2016). 19, 720–730. doi: 10.1016/j.chom.2016.03.010 27066744 PMC4866885

[37] Rashid MU, Lao Y, Spicer V, Coombs KM. Zika Virus Infection of Sertoli Cells Alters Protein Expression Involved in Activated Immune and Antiviral Response Pathways, Carbohydrate Metabolism and Cardiovascular Disease. Viruses (2022). 14. doi: 10.3390/v14020377 35215967 PMC8878972

[38] Rashid MU, Zahedi-Amiri A, Glover KKM, Gao A, Nickol ME, Kindrachuk J, Wilkins JA, Coombs KM. Zika virus dysregulates human Sertoli cell proteins involved in spermatogenesis with little effect on tight junctions. PLoS neglected tropical diseases (2020). 14, e0008335. doi: 10.1371/journal.pntd.0008335 32511241 PMC7279580

[39] Mayère C, Regard V, Perea-Gomez A, Bunce C, Neirijnck Y, Djari C, Bellido-Carreras N, Sararols P, Reeves R, Greenaway S., et al. Origin, specification and differentiation of a rare supporting-like lineage in the developing mouse gonad. Science advances (2022). 8, eabm0972. doi: 10.1126/sciadv.abm0972 35613264 PMC10942771

[40] Kumar A, Jovel J, Lopez-Orozco J, Limonta D, Airo AM, Hou S, Stryapunina I, Fibke C, Moore RB, Hobman TC. Human Sertoli cells support high levels of Zika virus replication and persistence. Sci Rep (2018). 8, 5477. doi: 10.1038/s41598-018-23899-x 29615760 PMC5883016

[41] de Jong J, Stoop H, Gillis AJM, Hersmus R, van Gurp RJHLM, van de Geijn G-JM, van Drunen E, Beverloo HB, Schneider DT, Sherlock JK, et al. Further characterization of the first seminoma cell line TCam-2. Genes, Chromosomes and Cancer (2008). 47, 185–196. doi: 10.1002/gcc.20520 18050305

[42] Young JC, Jaiprakash A, Mithraprabhu S, Itman C, Kitazawa R, Looijenga LH, Loveland KL. TCam-2 seminoma cell line exhibits characteristic foetal germ cell responses to TGF-beta ligands and retinoic acid. Int J Androl (2011). 34, e204–217. doi: 10.1111/j.1365-2605.2011.01170.x 21668453

[43] Klein B, Schuppe HC, Bergmann M, Hedger MP, Loveland BE, Loveland KL An in vitro model demonstrates the potential of neoplastic human germ cells to influence the tumour microenvironment. Andrology (2017). 5, 763–770. 28544640 10.1111/andr.12365

[44] Garcia-Minambres A, Eid SG, Mangan NE, Pade C, Lim SS, Matthews AY, de Weerd NA, Hertzog PJ, Mak J. Interferon epsilon promotes HIV restriction at multiple steps of viral replication. Immunol Cell Biol (2017). 95, 478–483. doi: 10.1038/icb.2016.123 28045025

[45] de Geus ED, Volaric JS, Matthews AY, Mangan NE, Chang J, Ooi JD, de Weerd NA, Giles EM, Hertzog PJ. Epithelially Restricted Interferon Epsilon Protects Against Colitis. Cellular and molecular gastroenterology and hepatology (2023). 17, 267–278. doi: 10.1016/j.jcmgh.2023.10.006 37879406 PMC10765064

[46] Nicolas N, Michel V, Bhushan S, Wahle E, Hayward S, Ludlow H, de Kretser DM, Loveland KL, Schuppe HC, Meinhardt A., et al. Testicular activin and follistatin levels are elevated during the course of experimental autoimmune epididymo-orchitis in mice. Sci Rep (2017). 7, 42391. doi: 10.1038/srep42391 28205525 PMC5304336

[47] Wijayarathna R, Pasalic A, Nicolas N, Biniwale S, Ravinthiran R, Genovese R, Muir JA, Loveland KL, Meinhardt A, Fijak M, Hedger MP. Region-specific immune responses to autoimmune epididymitis in the murine reproductive tract. Cell Tissue Res (2020). 381, 351–360. doi: 10.1007/s00441-020-03215-8 32383098

[48] Wijayarathna R, Hedger MP. New aspects of activin biology in epididymal function and immunopathology. Andrology. (2023). doi: 10.1111/andr.13523 37644728

[49] Knight PG, Muttukrishna S, Groome NP. Development and application of a two-site enzyme immunoassay for the determination of ’total’ activin-A concentrations in serum and follicular fluid. Journal of Endocrinology (1996). 148, 267–279. doi: 10.1677/joe.0.1480267 8699141

[50] Livak KJ, Schmittgen TD. Analysis of relative gene expression data using real-time quantitative PCR and the 2(-Delta Delta C(T)) Method. Methods (San Diego, Calif.) (2001). 25, 402–408. doi: 10.1006/meth.2001.1262 11846609

[51] Team RC. R: A language and environment for statistical computing. R Foundation for Statistical Computing, Vienna, Austria. (2021).

[52] Tian L, Su S, Dong X, Amann-Zalcenstein D, Biben C, Seidi A, Hilton DJ, Naik SH, Ritchie ME. scPipe: A flexible R/Bioconductor preprocessing pipeline for single-cell RNA-sequencing data. PLOS Computational Biology (2018). 14, e1006361. doi: 10.1371/journal.pcbi.1006361 30096152 PMC6105007

[53] Liao Y, Smyth GK, Shi W. The R package Rsubread is easier, faster, cheaper and better for alignment and quantification of RNA sequencing reads. Nucleic acids research (2019). 47, e47. doi: 10.1093/nar/gkz114 30783653 PMC6486549

[54] Durinck S, Moreau Y, Kasprzyk A, Davis S, De Moor B, Brazma A, Huber W. BioMart and Bioconductor: a powerful link between biological databases and microarray data analysis. Bioinformatics (2005). 21, 3439–3440. doi: 10.1093/bioinformatics/bti525 16082012

[55] Durinck S, Spellman PT, Birney E, Huber W. Mapping identifiers for the integration of genomic datasets with the R/Bioconductor package biomaRt. Nature protocols (2009). 4, 1184–1191. doi: 10.1038/nprot.2009.97 19617889 PMC3159387

[56] Robinson MD, McCarthy DJ, Smyth GK. edgeR: a Bioconductor package for differential expression analysis of digital gene expression data. Bioinformatics (2010). 26, 139–140. doi: 10.1093/bioinformatics/btp616 19910308 PMC2796818

[57] Robinson MD, Oshlack A. A scaling normalization method for differential expression analysis of RNA-seq data. Genome Biology (2010). 11, R25. doi: 10.1186/gb-2010-11-3-r25 20196867 PMC2864565

[58] Law CW, Chen Y, Shi W, Smyth GK. voom: precision weights unlock linear model analysis tools for RNA-seq read counts. Genome Biology (2014). 15, R29. doi: 10.1186/gb-2014-15-2-r29 24485249 PMC4053721

[59] Ritchie ME, Phipson B, Wu D, Hu Y, Law CW, Shi W, Smyth GK. limma powers differential expression analyses for RNA-sequencing and microarray studies. Nucleic acids research (2015). 43, e47. doi: 10.1093/nar/gkv007 25605792 PMC4402510

[60] Phipson B, Lee S, Majewski IJ, Alexander WS, Smyth GK. Robust hyperparameter estimation protects against hypervariable genes and improves power to detect differential expression. The annals of applied statistics (2016). 10, 946–963. doi: 10.1214/16-AOAS920 28367255 PMC5373812

[61] McCarthy DJ, Smyth GK. Testing significance relative to a fold-change threshold is a TREAT. Bioinformatics (2009). 25, 765–771. doi: 10.1093/bioinformatics/btp053 19176553 PMC2654802

[62] Liberzon A, Birger C, Thorvaldsdóttir H, Ghandi M, Mesirov JP, Tamayo P. The Molecular Signatures Database (MSigDB) hallmark gene set collection. Cell systems (2015). 1, 417–425. doi: 10.1016/j.cels.2015.12.004 26771021 PMC4707969

[63] Milacic M, Beavers D, Conley P, Gong C, Gillespie M, Griss J, Haw R, Jassal B, Matthews L, May B, et al. The Reactome Pathway Knowledgebase 2024. Nucleic acids research (2023). 52, D672–D678. 10.1093/nar/gkad1025 PMC10767911 37941124

[64] Subramanian A, Tamayo P, Mootha VK, Mukherjee S, Ebert BL, Gillette MA, Paulovich A, Pomeroy SL, Golub TR, Lander ES, Mesirov JP. Gene set enrichment analysis: a knowledge-based approach for interpreting genome-wide expression profiles. Proc Natl Acad Sci U S A (2005). 102, 15545–15550. doi: 10.1073/pnas.0506580102 16199517 PMC1239896

[65] Wu D, Smyth GK. Camera: a competitive gene set test accounting for inter-gene correlation. Nucleic acids research (2012). 40, e133–e133. doi: 10.1093/nar/gks461 22638577 PMC3458527

[66] Ma W, Li S, Ma S, Jia L, Zhang F, Zhang Y, Zhang J, Wong G, Zhang S, Lu X, et al. Zika Virus Causes Testis Damage and Leads to Male Infertility in Mice. Cell (2016). 167, 1511–1524 e1510. doi: 10.1016/j.cell.2016.11.016 27884405

[67] Jiang Q, Wang F, Shi L, Zhao X, Gong M, Liu W, Song C, Li Q, Chen Y, Wu H, Han, D. C-X-C motif chemokine ligand 10 produced by mouse Sertoli cells in response to mumps virus infection induces male germ cell apoptosis. Cell Death Dis (2017). 8, e3146. 29072682 10.1038/cddis.2017.560 PMC5680925

[68] Klein B, Pant S, Bhushan S, Kautz J, Rudat C, Kispert A, Pilatz A, Wijayarathna R, Middendorff R, Loveland KL., et al. Dexamethasone improves therapeutic outcomes in a preclinical bacterial epididymitis mouse model. Human reproduction (Oxford, England) (2019). 34, 1195–1205. doi: 10.1093/humrep/dez073 31211847

[69] Liu WH, Wang F, Yu XQ, Wu H, Gong ML, Chen R, Zhang WJ, Han RQ, Liu AJ, Chen YM, Han DS. Damaged male germ cells induce epididymitis in mice. Asian journal of andrology (2020). 22, 472–480. doi: 10.4103/aja.aja_116_19 31696835 PMC7523604

