# Supplementary figures and images for: Interferon epsilon is produced in the testis and protects the male reproductive tract against virus infection, inflammation and damage
Source: PLoS Pathog. 2024 Dec 2;20(12):e1012702. doi: 10.1371/journal.ppat.1012702 (PMC11637430; doi:10.1371/journal.ppat.1012702)

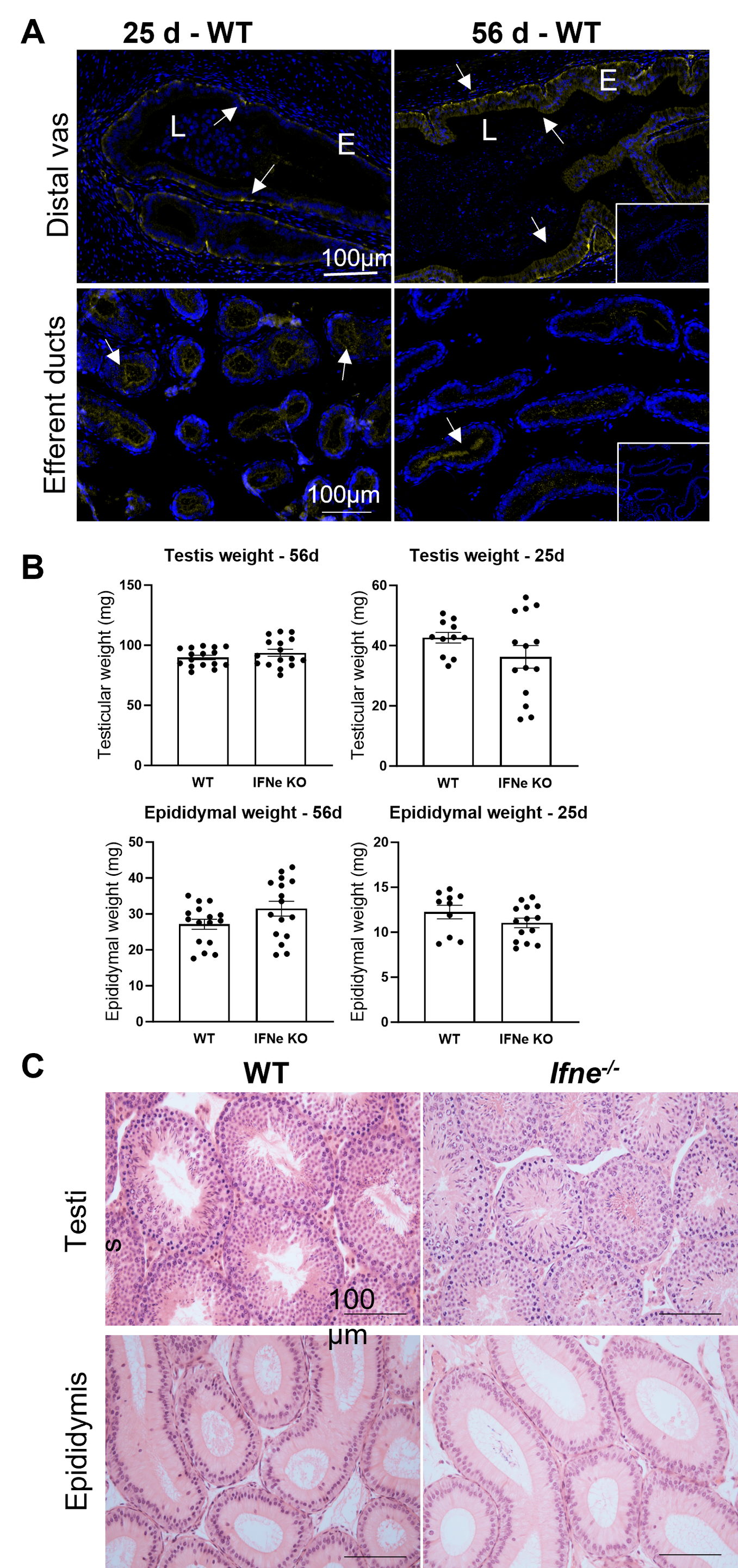

Supplement: S1 Fig — (A) Immunofluorescence staining for IFNɛ in the distal region of the WT mouse vas deferens and efferent ducts at 56 and 25 days of age. Scale bars = 50 μm. (B) Testicular and epididymal weights at 25 and 56 days of age in Ifne-/- mice compared to WT controls. (C) Testicular and epididymal histology of Ifne-/- mice compared to WT controls at 56 days of age. Representative images from n = 4 mice per genotype. Scale bars = 100μm. (TIF) [file ppat.1012702.s001.tif]

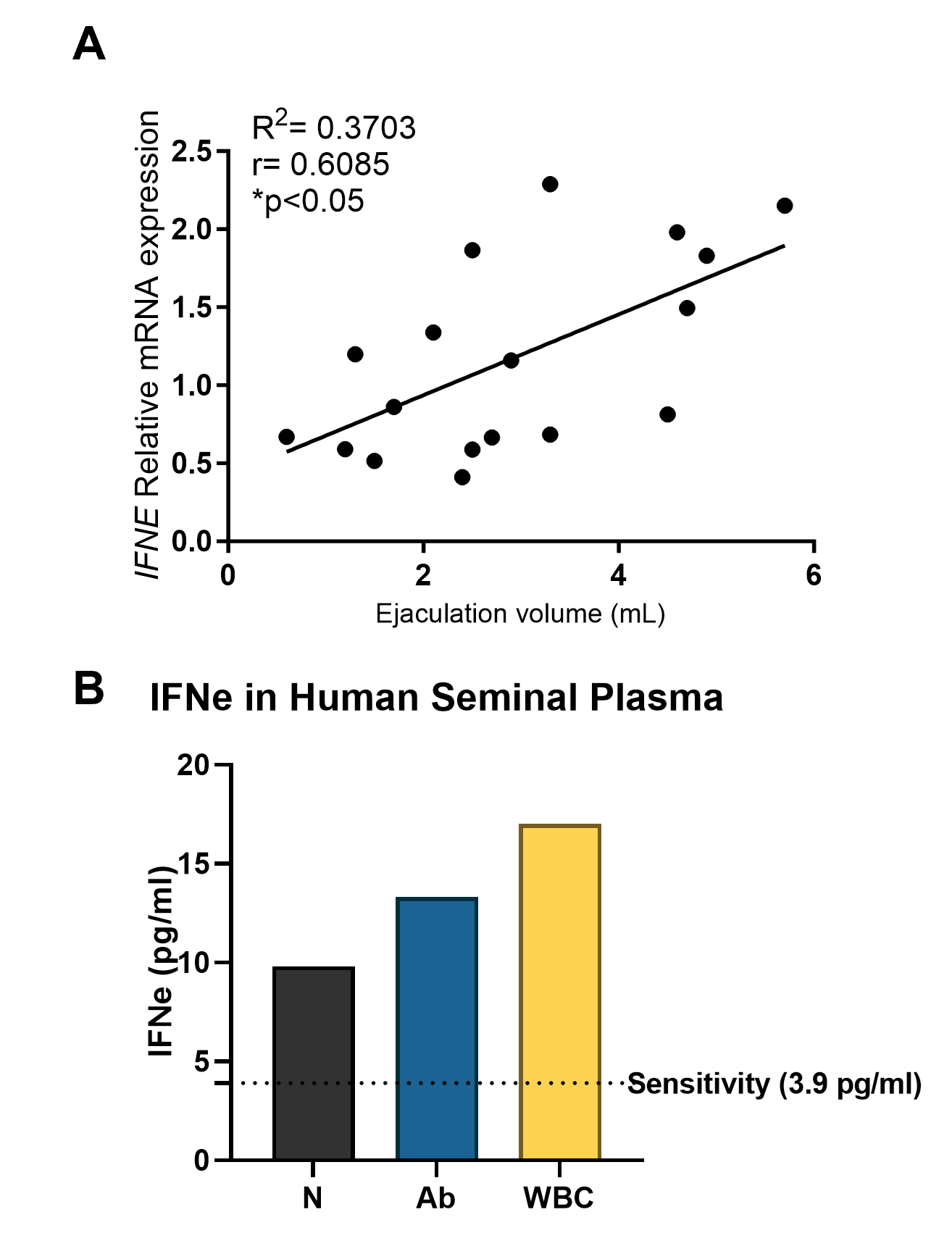

Supplement: S2 Fig — (A) Correlation between IFNɛ mRNA measured by qRT-PCR in testicular tissue samples and ejaculation volume in a cohort of infertile men. (B) IFNɛ protein measured using a two-site ELISA assay in human seminal plasma from normozoospermic men (N), patients with antibodies (Ab) and patients with leukocytospermia (WBC) (Limit of detection/Assay sensitivity: 3.9 pg/ml. Pooled samples, 25μl testicular interstitial fluid per patient from 4 patients in each category). (TIF) [file ppat.1012702.s002.tif]

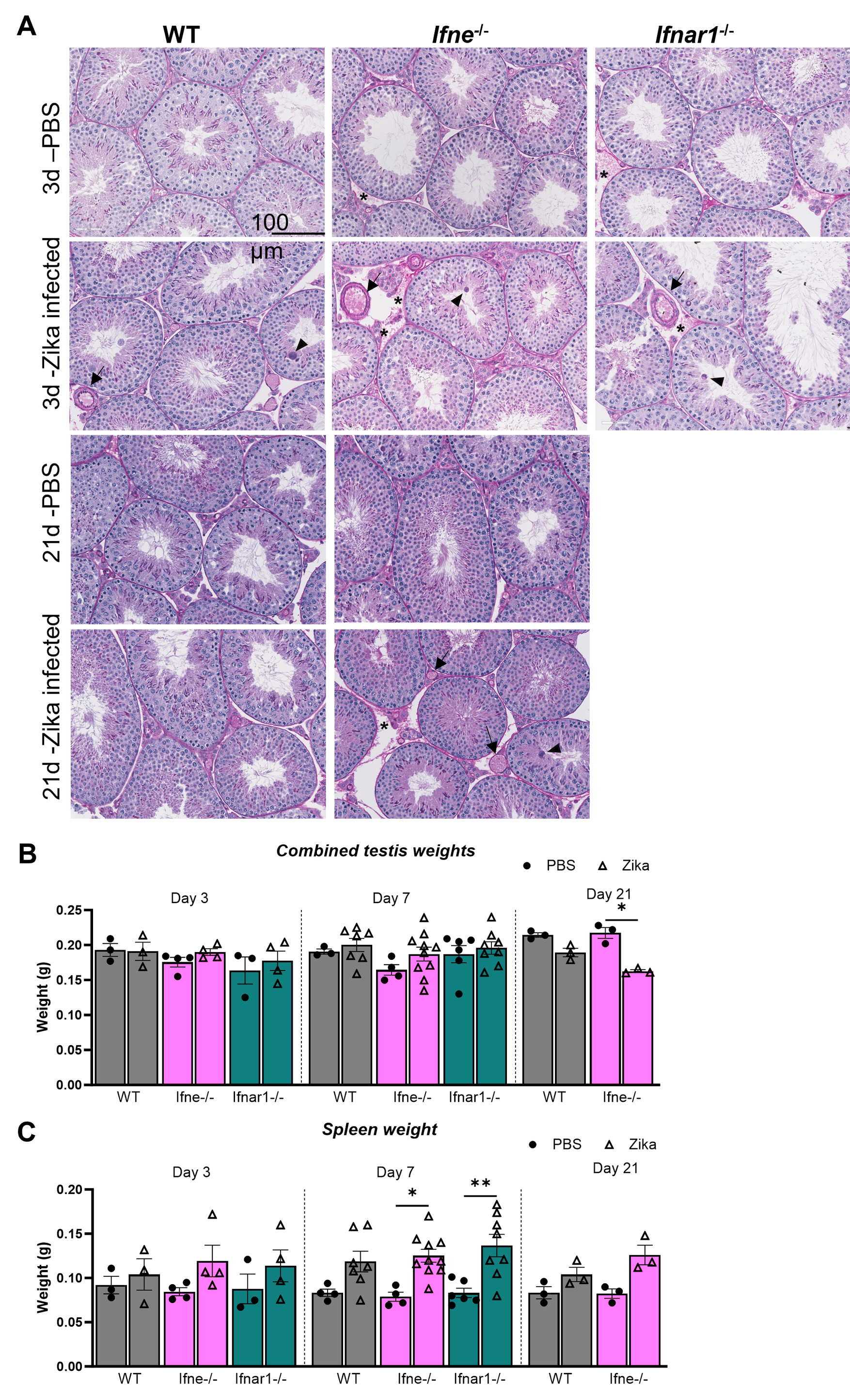

Supplement: S3 Fig — (A) Vascular congestion (arrows), oedema (asterisk) and degenerating germ cells (arrowheads). (B) Combined weights of right and left testes (in mg) 3, 7 and 21 days post-Zika infection. (C) Spleen weight (mg) 3, 7 and 21 days post-Zika infection. Ifnar1-/- mice were only examined at 3 and 7 days due to ethical limitations. Representative images from n = 3 mice per experimental group. Scale bars = 100μm. (TIF) [file ppat.1012702.s003.tif]

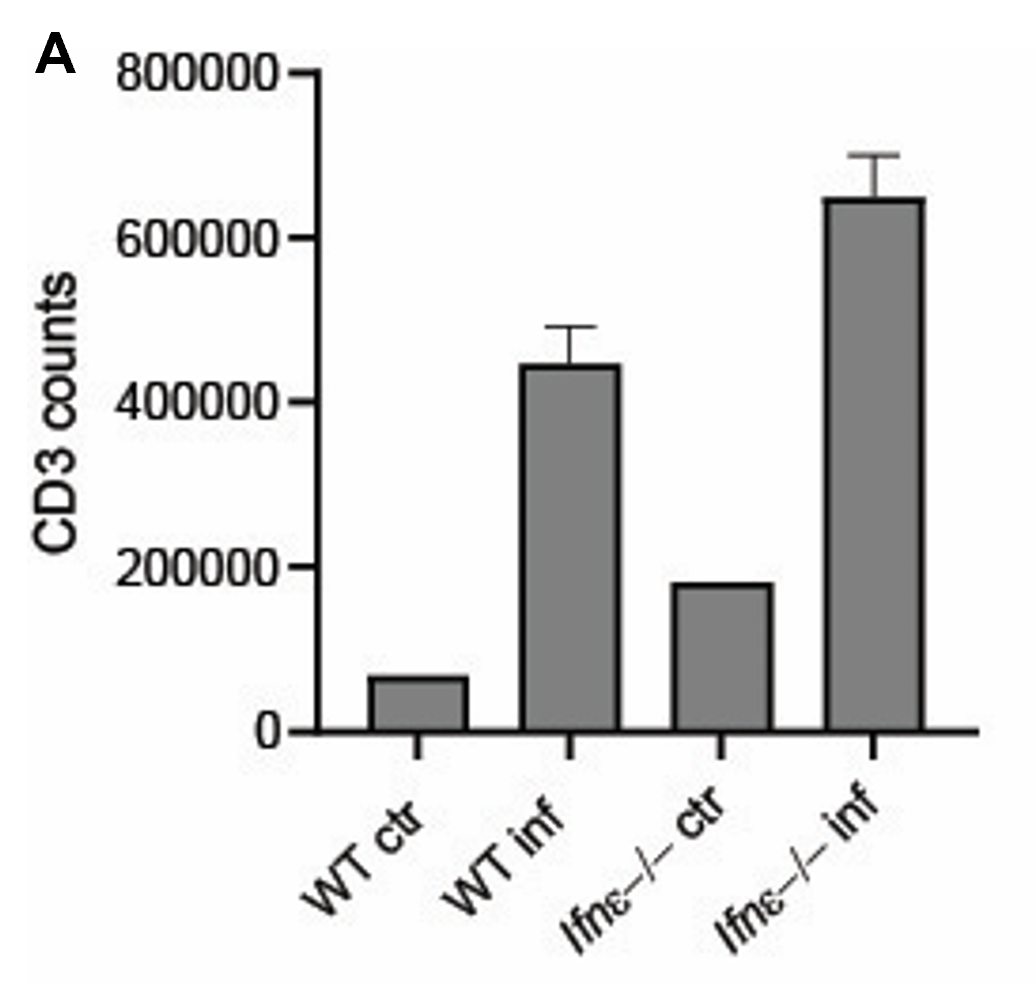

Supplement: S4 Fig — CD3 T cells counts in PBS injected WT controls, Zika infected WT, PBS injected Ifne-/- controls and Zika infected Ifne-/- mice Data are presented as mean ± SD. (TIF) [file ppat.1012702.s004.tif]

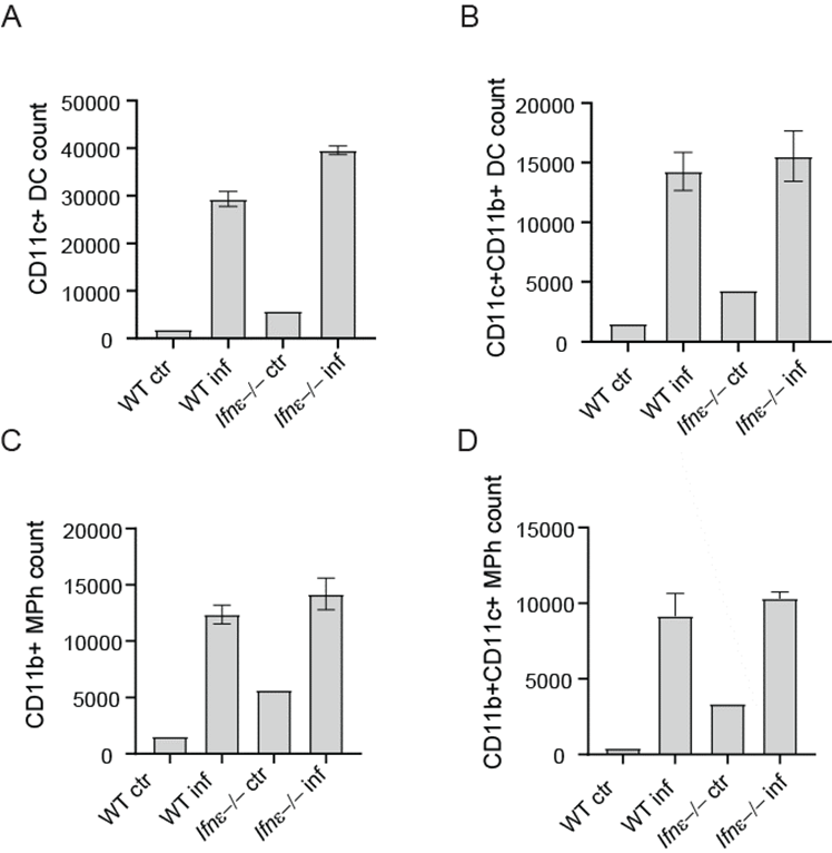

Supplement: S5 Fig — (A) CD11c+ and (B) CD11c+CD11b+ dendritic cell (DC) counts. (C) CD11b+ and (D) CD11b+CD11c+ Macrophage (MPh) counts. MPh were defined as F4/80+CD11b+CD11c±. Data are presented as mean ± SD. (TIF) [file ppat.1012702.s005.tif]

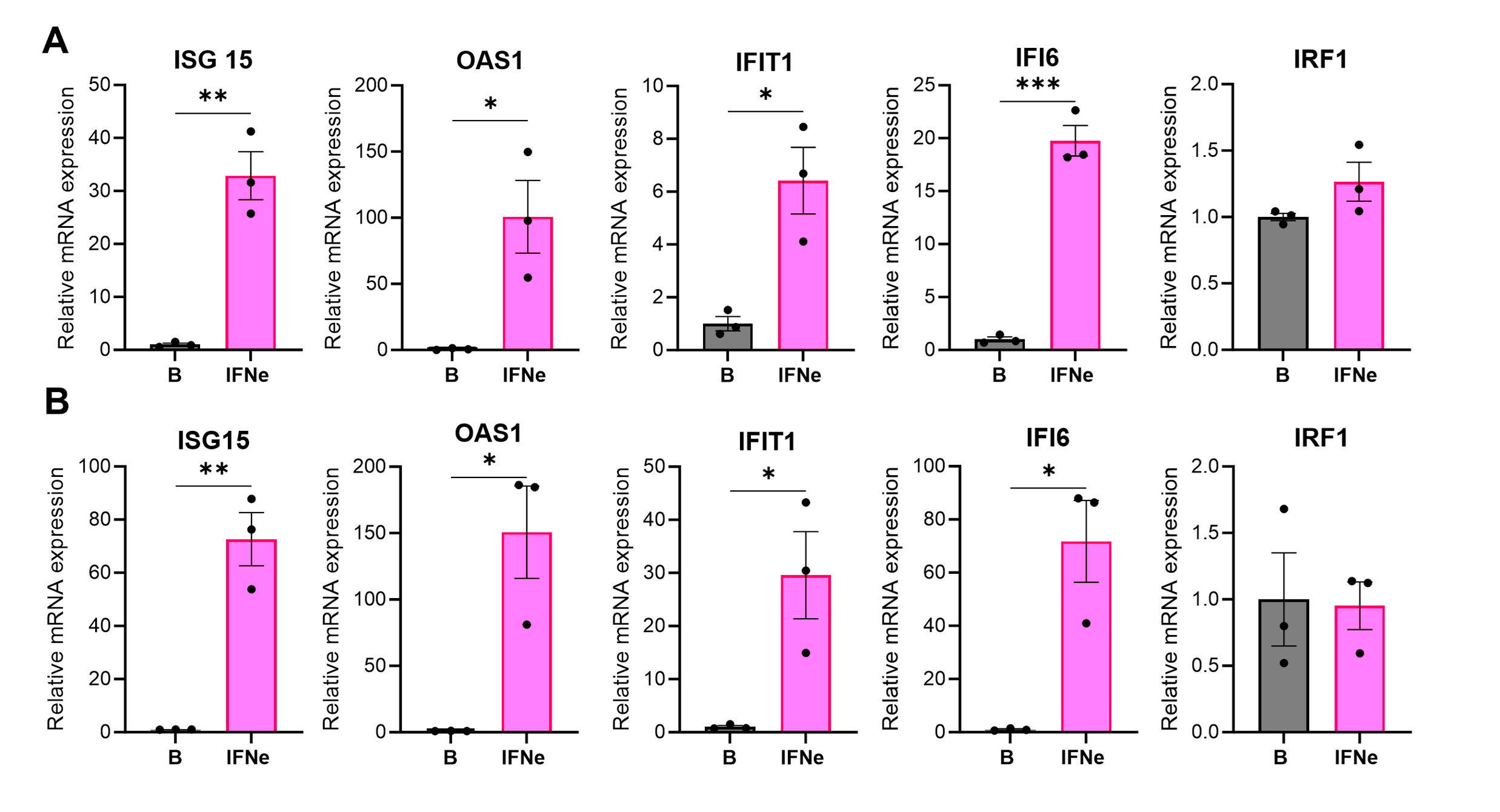

Supplement: S6 Fig — ISGs induced 12 hours post-treatment by 100 IU exogenous IFNɛ, in an uninfected (A) human seminoma cell line (TCam-2) and (B) human Leydig cells. All genes were normalised to the housekeeping gene RPLP0, B = Buffer treated cultures, IFNɛ = IFNɛ treated cultures. Each individual data point in the graphs represent the average of three technical replicates per culture round. One-Way ANOVA to compare more than 2 data sets, Student’s t-test to compare 2 data sets, *p < 0.05, **p<0.01, ***p< 0.001. (TIF) [file ppat.1012702.s006.tif]

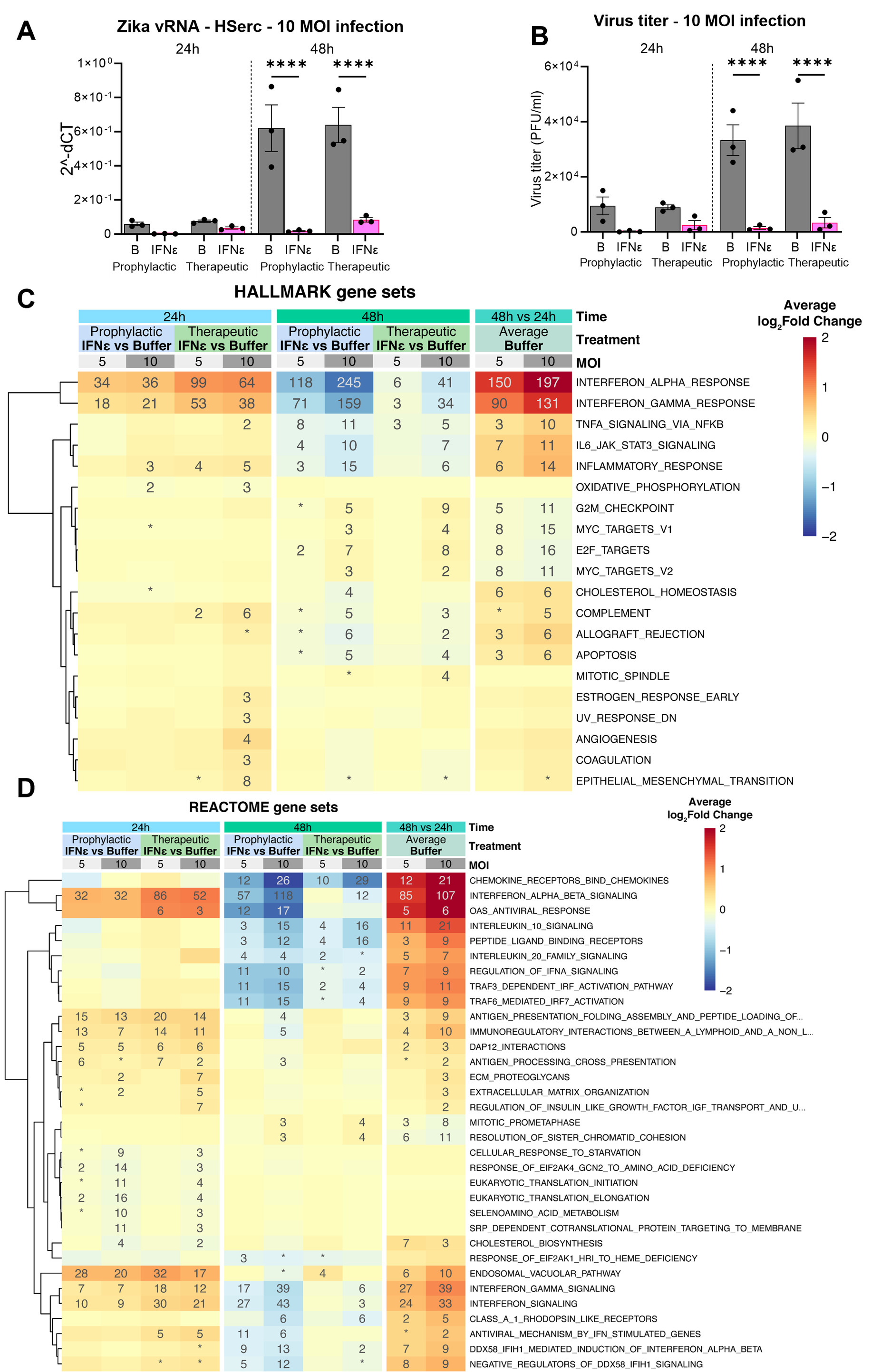

Supplement: S7 Fig — (A) qRT-PCR detection of Zika virus RNA in primary human Sertoli cell cultures (HSerc) treated with 100 U/ml rhIFNɛ or buffer either before or after infection with 10 MOI Zika virus, assayed 24 and 48 hours post-infection (2^-dCt = relative copy number) (B) Infectious viral burden in Sertoli cell culture media measured by plaque assays 24 and 48 hours post-infection with 10 MOI Zika virus B = Buffer treated cultures, IFNɛ = IFNɛ treated cultures. Each individual data point in the graphs represent the average of three technical replicates per culture round. 2-Way ANOVA, *P < 0.05, **P < 0.01***P < 0.001. (C–D) Heat maps showing some of the most significant gene sets from the MsigDB Hallmark (C) and Reactome (D) gene set collections, associated with the RNA-seq analysis of human Sertoli cells, with each rhIFNɛ treatment compared to its matched buffer control, assessed using the cameraPR function. The colours indicate the average log2 fold changes of all genes in the gene set for each comparison, with the scale truncated to ±2. The significance of each gene set is Indicated by the text as the −log10 FDR-adjusted p-value. * denotes adjusted p-value threshold p < 0.05, 2 denotes p < 0.01, 3 denotes p < 0.001 and so on. (TIF) [file ppat.1012702.s007.tif]

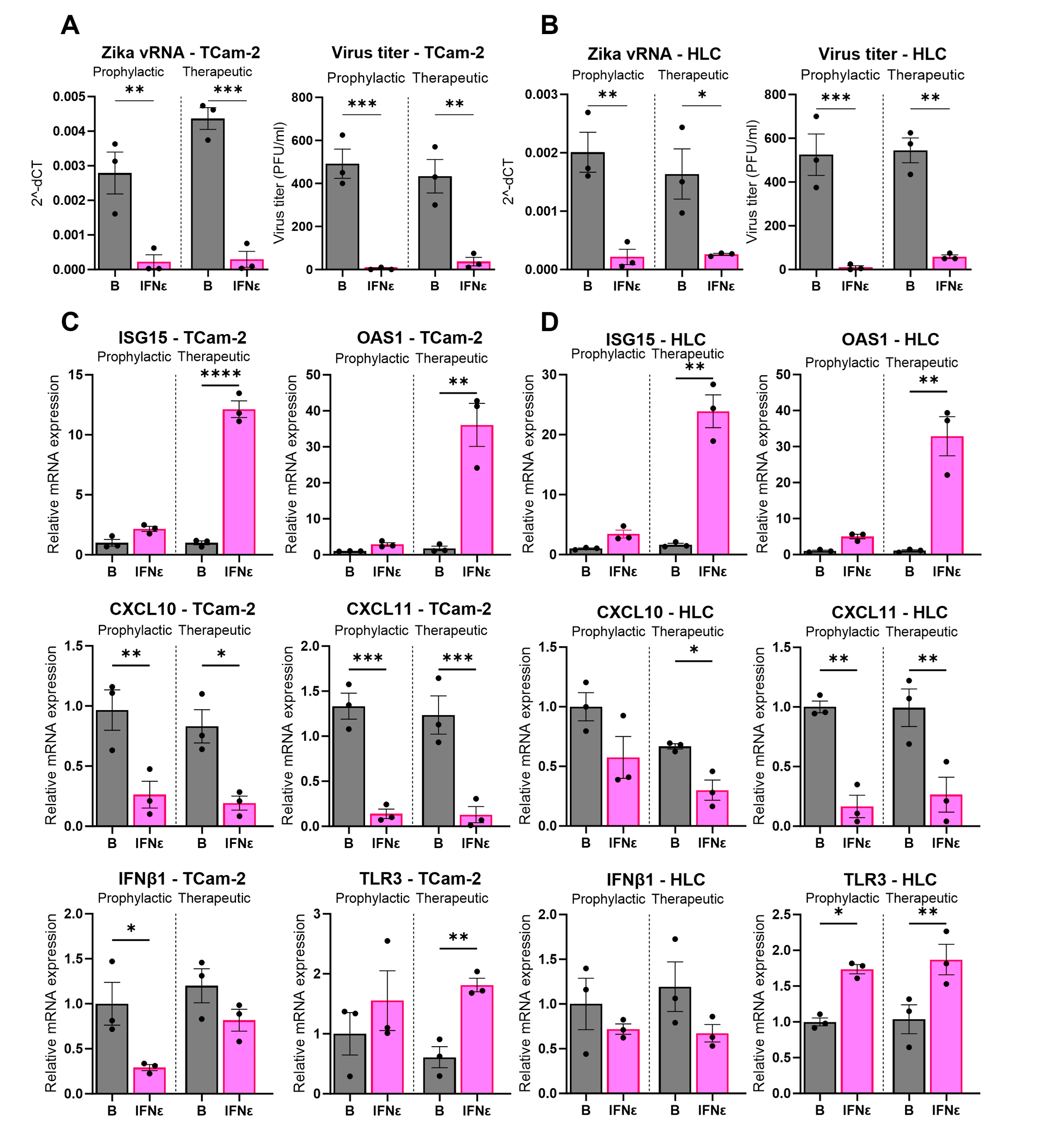

Supplement: S8 Fig — qRT-PCR detection of Zika virus RNA, and infectious viral burden measured by plaque assays in (A) a human Seminoma cell line, and (B) primary human Leydig cell cultures (HLC) treated with 100 U/ml rhIFNɛ or buffer either before (prophylactic) or after (therapeutic) infection with 5 MOI Zika virus, assayed 24 hours post-infection (2^-dCt = relative copy number). (C) qRT-PCR of anti-viral effectors, interferons, pro-inflammatory genes, and pattern recognition receptor genes from TCam-2 cells and (D) primary human Leydig cell cultures infected with 5 MOI Zika virus at 24 hours post-infection. B = Buffer treated cultures, IFNɛ = IFNɛ treated cultures. Each individual data point in the graphs represent the average of three technical replicates per culture round. One-Way ANOVA to assess data sets with one variable, 2-Way ANOVA for 2 variables. *P < 0.05, **P < 0.01, ***P < 0.001, ****P < 0.0001. (TIF) [file ppat.1012702.s008.tif]

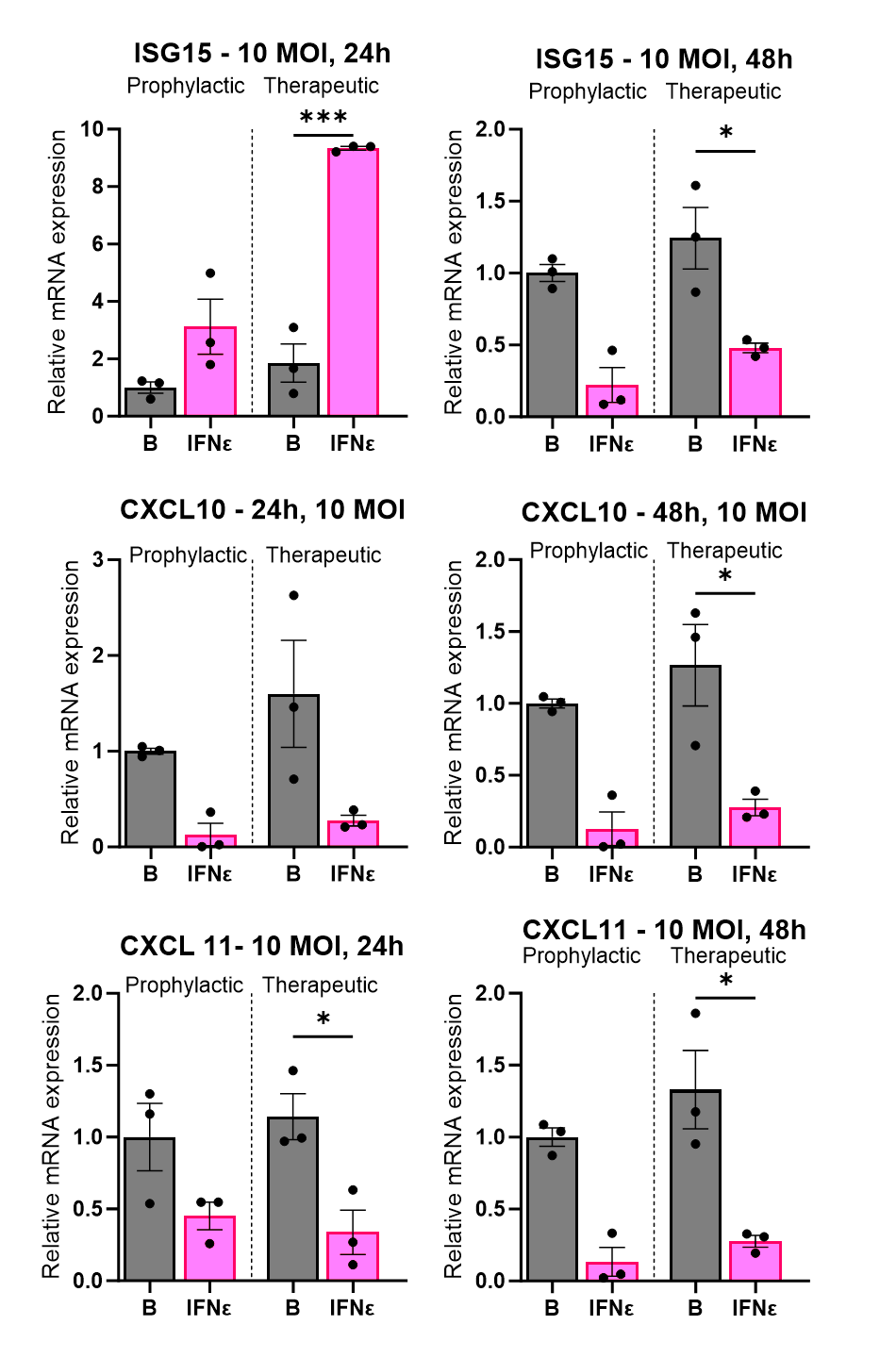

Supplement: S9 Fig — B = Buffer treated cultures, IFNɛ = IFNɛ treated cultures. Each individual data point in the graphs represent the average of three technical replicates per culture round. 2-way ANOVA, *P < 0.05.) (TIF) [file ppat.1012702.s009.tif]
