# Supplementary material for: Interferon epsilon is produced in the testis and protects the male reproductive tract against virus infection, inflammation and damage
Source: PLoS Pathog. 2024 Dec 2;20(12):e1012702. doi: 10.1371/journal.ppat.1012702 (PMC11637430; doi:10.1371/journal.ppat.1012702)
Supplement: S1 Table — (PDF) [file ppat.1012702.s010.pdf]

S1 Table. qRT-PCR primers used in the study.

Gene/ Target	Species	Sequence (5'– 3')
RPLP0	Human	F - AGA TGC AGC AGA TCC GCAT<br>R - GGA TGG CCT TGC GCA
IFNE	Human	F - AGGACACACTCTGGCCATTC<br>R - CTCCCAACCATCCAGAGAAA
IFNB1	Human	F - TGCTCTGGCACAACAGGTAG<br>R - CAGGAGAGCAATTTGGAGGA
IFNAR1	Human	F - AGTGCCTCCACGCTTTTTA<br>R - GCTTGTACGCGGAGAAGGTA
IFNAR2	Human	F - CAAGCACCATAGTGACACTGAAA<br>R - CCATATCCATGGCTTCCAAC
IFNL1	Human	F - GGA AGA GTC ACT CAA GCT GAA AAA C<br>R - AGA AGC CTC AGG TCC CAA TTC
ISG15	Human	F - TGG CGG GCA ACG AAT T<br>R - GGG TGA TCT GCG CCT TCA
OAS1	Human	F - CCGTGAAGTTTGAGGTCCAG<br>R - GGTTTATAGCCGCCAGTCAA
IFIT1	Human	F - AAC TTA ATG CAG GAA GAA CAT GAC AA<br>R - CTG CCA GTC TGC CCA TGT G
TLR3	Human	F - CCTGGTTTGTTAATTGGATTAACGA<br>R - TGAGGTGGAGTGTTGCAAAGG
TLR9	Human	F - GAAGGGACCTCGAGTGTGAA<br>R - GCCTGCACCAGGAGAGAGACAG
CXCL10	Human	F - TCC ACG TGT TGA GAT CAT TGC<br>R - TCT TGA TGG CCT TCG ATT CTG
CXCL11	Human	F - CCT TGG CTG TGA TAT TGT GTG C<br>R - CCA CTT TCA CTG CTT TTA CCC C
IRF1	Human	F - CCA GCC CTG ATA CCT TCT CTG A<br>R - AAG TCC TGC ATG TAG CCT GGA A
IFI6	Human	F - CTG AAG ATT GCT TCT CTT CTC<br>R - CAC TTT TTC TTA CCT GCC TC
Rplp0	Murine	F - GGA CCC GAG AAG ACC TCC TT<br>R - GCA CAT CAC TCA GAA TTT CAA TGG
Ifne	Murine	F - GAA ACG GAT TCC CTT CCA AT<br>R - ACT GCT GGA CTG ACG AGC TT
Ifnb1	Murine	F - AGA AAG GAC GAA CAT TCG GAA A<br>R - CCG TCA TCT CCA TAG GGA TCT T
Ifnar1	Murine	F - GCAGTGTGACCTTTTCAGCA<br>R - GAGAATTCACACTTGGTCGTTG
Ifnar2	Murine	F - CCCACCCGCTAACTACACC<br>R - GACGACGTAGCTCTCCATGC
Isg15	Murine	F - TGAGAGCAAGCAGCCAGAAG<br>R - ACGGACACCAGGAAATCGTT
Casp1	Murine	F - CGCCATGGCTGACAAGATCCTG<br>R - GGTCCCGTGCCTTGTCCATAGC
Cyp11a1	Murine	F - ACATGGCCAAGATGGTACAGTTG<br>R - ACGAAGCACCAGGTCATTCAC
Cyp17a1	Murine	F - TGACCAGTATGTAGGCTTCAGTCG<br>R - TCCTTCGGGATGGCAAACTCTC-
Star	Murine	F - CGGGTGGATGGGTCAAGTTC<br>R - CCAAGCGAAACACCTTGCC

Inha	Murine	F - ATGCACAGGACCTCTGAACC<br>R - GGAGAACGGGTATGTGGAGA
Tnp1	Murine	F - GCATGAGGAGAGGCAAGAAC<br>R - ATTGCGACTTGCATCATCGCCC
Sycp3	Murine	F - AGCCAATCAGCAGAGAGCTTG<br>R - CTTTAGATGTTTGCTCAGCGG
Cd45	Murine	F - ATGGTCCTCTGAATAAAGCCCA<br>R - TCAGCACTATTGGTAGGCTCC
F4/80	Murine	F - AGCACCATGTTAGCTGCTCT<br>R - GGGGCCCCTGTAGATACTGA
MHCII	Murine	F - TGGCCTTTTCATCCGTCACA<br>R - ACTGGCAGTCAGGAATTCGG
CD206	Murine	F - GGAAGCCCATTCCGGTATCT<br>R - CATCGCTTGCTGAGGGAATG
CD80	Murine	F - AGAGCCAGGGTAGTGCTAGG<br>R - GGCTCATGAGGTCTCATCTACCAT
CD86	Murine	F - TCTCCACGGAAACAGCATCTGA<br>R - ACTTACGGAAGCACCCACG
Cd14	Murine	F - GGCGCTCCGAGTTGTGACT<br>R - TACCTGCTTCAGCCCAGTGA
Asc	Murine	F - GACAGTACCAGGCAGTTCGT<br>R - AGTCCTTGCAGGTCAGGTTC
Nlrp3	Murine	F - CAGAGCCTACAGTTGGGTGA<br>R - TAGCAGTGAAGAGCAGTGCG
Casp1	Murine	F - ACGCCATGGCTGACAAGATCCTG<br>R - GGTCCCGTGCCTTGTCCATAGC
Inhba	Murine	F - ATCATCACCTTTGCCGAGTC<br>R - CTGAAATAGACGGATGGTGA
Inhbb	Murine	F - CGTCTCCGAGATCATCAGC<br>R - CAGGACATAGGGGAGCAGTT
Fst	Murine	F - AAAACCTACCGCAACGAATG<br>R - TTCAGAAGAGGAGGGCTCTG
Tgfb1	Murine	F – AGCCCGAAGCGGACTACTAT<br>R – TTCCACATGTTGCTCCACAC
Col1a1	Murine	F - ACGTCTGGTTTGGAGAGA<br>R - AGGAAGGTCAGCTGGATAG
Col1a2	Murine	F - TTCACCTACTCTGTCCTAGTC<br>R – CAGGCGAGATGGCTTATTT
Col4a1	Murine	F - TCAGGTCCACCTGGAATTA<br>R - GAAGTCCTTGAGAGCCTTTATC
Timp1	Murine	F – CCCCAGAAATCAACGAGAC<br>R – CTGGGACTTGTGGGCATATC
Mmp2	Murine	F – CAGGGAATGAGTACTGGGTCTATT<br>R - ACTCCAGTTAAAGGCAGCATCTAC
Mmp14	Murine	F - TTACAAGTGACAGGCAAGG<br>R – GCTTCCTCCGAACATTGG
Il6	Murine	F - ATGGATGCTACCAAACTGGAT<br>R - TGAAGGACTCTGGCTTTGTCT
Tnf	Murine	F - CAAATTCGAGTGACAAGCCTG<br>R - GAGATCCATGCCGTTGGC
ZIKV PRVABC59 – prM specific		F - GTG TGA TGC CAC CAT GAG CTA<br>R - TGG CAG GTT CCG TAC ACA AAC
