# Supplementary material for: Interferon epsilon is produced in the testis and protects the male reproductive tract against virus infection, inflammation and damage
Source: PLoS Pathog. 2024 Dec 2;20(12):e1012702. doi: 10.1371/journal.ppat.1012702 (PMC11637430; doi:10.1371/journal.ppat.1012702)
Supplement: S2 Table — (PDF) [file ppat.1012702.s011.pdf]

S2 Table. Histopathological damage scoring in mouse testis sections

Feature	Grade	Description
Interstitial edema	0	Absent
	1	Mild (1 - 10% of interstitial area affected on 3 consecutive histological sections of testis)
	2	Moderate (10 – 50% of interstitial area)
	3	Severe (>50% 50% of interstitial area)
Vascular congestion	0	Absent
	1	Mild (1 - 10% of testicular blood vessels appear congested on 3 consecutive histological sections of testis)
	2	Moderate (10 - 50% of blood vessels appear congested)
	3	Severe (>50% of blood vessels appear congested)
F4/80+ macrophages	0	No increase compared to uninfected WT
	1	Mild (1 - 10% increase of cell numbers in histological sections of testis, no cellular aggregates)
	2	Moderate (1 - 10% increase of cell numbers in histological sections of testis together with cellular aggregates in interstitium)
	3	Severe (>10% increase of cell numbers in histological sections of testis together with cellular aggregates in interstitium)
Caspase+ cells	0	No increase compared to uninfected WT
	1	Mild (1 - 10% increase of cell numbers in histological sections of testis)
	2	Moderate (1 - 10% increase of cell numbers in histological sections of testis)
	3	Severe (>10% increase of cell numbers in histological sections of testis)
