# Supplementary material for: Interferon epsilon is produced in the testis and protects the male reproductive tract against virus infection, inflammation and damage
Source: PLoS Pathog. 2024 Dec 2;20(12):e1012702. doi: 10.1371/journal.ppat.1012702 (PMC11637430; doi:10.1371/journal.ppat.1012702)
Supplement: S3 Table — (PDF) [file ppat.1012702.s012.pdf]

S3 Table. Histopathological damage scoring in mouse epididymis sections

Score	Description
0	Normal histology
1	Normal histology but > two-fold relative elevation of cytokine genes (*Tnf*, *II6*)
2	Intact epithelium but germ cell desquamation in lumen (1-2 cells per cross section)
3	Fibrosis, elevation of inflammatory genes, desquamated cells in lumen (>2 cells per cross section)
4	Reduction in ductal diameter and epithelial height, widespread structural damage due to epithelial desquamation, cytoplasmic vacuolation of epithelial cells, extensive immune infiltration and fibrosis
